# Photoreactions and Structural Changes of *Anabaena* Sensory Rhodopsin

**DOI:** 10.3390/s91209741

**Published:** 2009-12-03

**Authors:** Akira Kawanabe, Hideki Kandori

**Affiliations:** Department of Frontier Materials, Nagoya Institute of Technology, Showa-ku, Nagoya, 466-8555, Japan; E-Mail: kawanabe.akira@nitech.ac.jp (A.K.)

**Keywords:** photosensor, rhodopsin, photochromism, FTIR spectroscopy, UV-visible spectroscopy

## Abstract

*Anabaena* sensory rhodopsin (ASR) is an *archaeal*-type rhodopsin found in eubacteria. The gene encoding ASR forms a single operon with ASRT (ASR transducer) which is a 14 kDa soluble protein, suggesting that ASR functions as a photochromic sensor by activating the soluble transducer. This article reviews the detailed photoreaction processes of ASR, which were studied by low-temperature Fourier-transform infrared (FTIR) and UV-visible spectroscopy. The former research reveals that the retinal isomerization is similar to bacteriorhodopsin (BR), but the hydrogen-bonding network around the Schiff base and cytoplasmic region is different. The latter study shows the stable photoproduct of the all-*trans* form is 100% 13-*cis*, and that of the 13-*cis* form is 100% all-*trans*. These results suggest that the structural changes of ASR in the cytoplasmic domain play important roles in the activation of the transducer protein, and photochromic reaction is optimized for its sensor function.

## 1. Introduction

Photosynthesis is one of the most important chemical reactions in living cells because almost all energy spent by living things on Earth originates from it. Photosynthesis mainly takes place in the chloroplasts of plants, where a photoinduced electron transfer reaction first stores light energy, but eventually a proton gradient is formed across the membrane. The proton gradient is used for the synthesis of ATP, because it is a driving force of an enzyme ATP-synthase. Some bacteria directly convert light energy into a proton gradient through a proton pump. Light sensing is also important: Plants must avoid ultra-violet (UV) light, because it can possibly damage their genes. In addition, they have to sense orange or red light, as photosynthesis is more efficiency under these lights.

**Figure 1.**
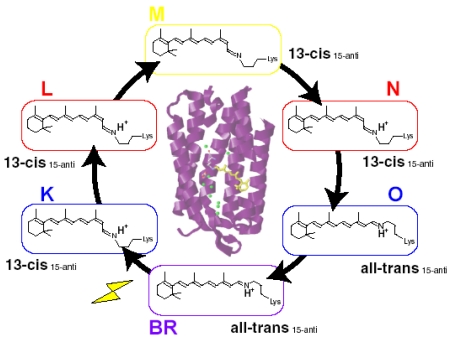
The bacteriorhodopsin photocycle (PDB code: 1C3W) [7]. The reaction starts with light and returns to the initial state through the various intermediates within 10 ms.

**Figure 2.**
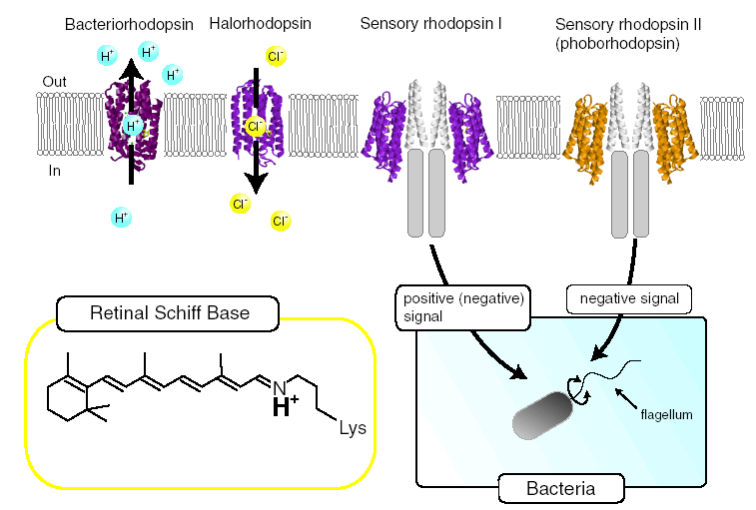
There are four archaeal rhodopsins in *Halobacterium salinarum*: bacteriorhodopsin, halorhodopsin, sensory rhodopsin I and II. All of them have seven transmembrane helices and an all-*trans* retinal as a chromophore.

Four archaeal type rhodopsins [Bacteriorhodopsin (BR), Halorhodopsin (HR), Sensory rhodopsin I (SRI), and Sensory rhodopsin II (SRII); also called phoborhodopsin (pR)] were discovered in the cytoplasmic membrane of *Halobacterium salinarum* [1-4]. The former two (BR and HR) function as light-driven proton and chloride pumps, respectively, while the latter two (SRI and SRII) are responsible for attractive and repellent phototaxis, respectively (Figure 1). They have a retinal molecule as a chromophore, which forms a Schiff base linkage with a lysine residue of the 7th helix. An all-*trans* form (all-*trans*, 15-*anti*) is the functional form in BR, HR, SRI and SRII, and absorption of light leads to isomerization to the 13-*cis*, 15-*anti* form, which triggers protein structural changes for function. In the case of BR, a cyclic reaction comprises the series of intermediates, K, L, M, N, and O (Figure 2) [5,6]. During the photocycle, a proton is transported from the cytoplasmic to the extracellular side. They have been extensively studied as model systems converting light energy to chemical potential or environmental signals. Although such archaeal type rhodopsins were considered to exist only in Archaea, during the last decade the various genome sequencing projects have revealed that archaeal rhodopsins also exist in Eukaryota and Bacteria. In eucaryotes, archaeal rhodopsins have been found in fungi [8], green algae [9,10], dinoflagellates [11], and cryptomonads [12].

**Figure 3.**
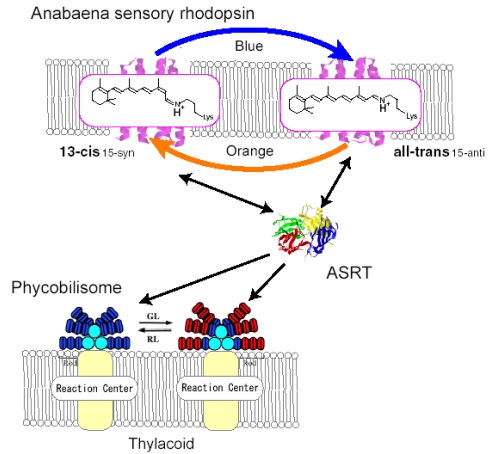
Proposed function of *Anabaena* sensory rhodopsin (1XIO[16]). ASR is interconverted into two isomeric states, which have different interactions with ASRT (2II9) [17]. ASRT maybe controls the expression level of phycobilisome proteins (phycocyanin and phycoerythrin) [15]. Phycobilisome graphic is from Grossman *et al.* [18]

Eubacterial rhodopsins were found both in γ- and α-proteobacteria [13,14] as well as in *Anabaena* (*Notstoc*) sp. PCC7120, a freshwater cyanobacterium [15], which was called *Anabaena* Sensory Rhodopsin (ASR). The gene encoding ASR, which is a membrane protein of 261 residues (26 kDa), and a smaller gene encoding a soluble protein of 125 residues (14 kDa), which is called ASRT (ASR transducer), are under the same promoter in a single operon [15]. The opsin expressed heterologously in *Escherichia coli* membranes binds all-*trans* retinal to form a pink pigment (λ_max_ = 549 nm) with a photochemical reaction cycle half-life of 110 ms (pH 6.8, 18 °C) [15]. The previous study revealed that co-expression with ASRT increased the rate of the photocycle, indicating physical interaction with ASR and the possibility that ASR works as a photosensor protein (Figure 3) [15]. It should be noted that SRI and SRII activate transmembrane transducer proteins (Figure 1). In this sense, ASR is closer to visual rhodopsins that activate soluble G-proteins.

**Figure 4.**
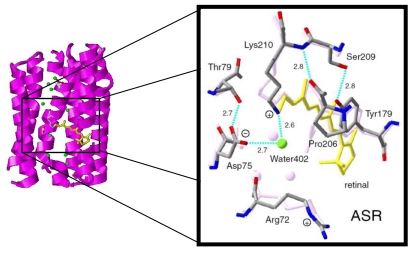
(Left) X-ray crystallographic structure of ASR (1XIO[16]). Purple ribbons, green spheres, yellow and white sticks correspond to helices, water molecules, retinal and amino acid residues, respectively. (Right) The Schiff base region of ASR and BR (translucent structure, 1C3W [7]), respectively. Each retinal molecule between ASR and BR is fitted to compare the hydrogen-bonding networks by using Swiss-PdbViewer [19]. Top and bottom regions correspond to the cytoplasmic and extracellular sides. The green sphere (Water 402) represents a water molecule which forms a hydrogen bond bridge between the protonated Schiff base and its counterion, Asp75. Hydrogen-bonds (blue dashed lines) are inferred from the structure and the numbers are the hydrogen-bond distances in Å. This figure is adapted with permission from Furutani *et al.* [20]. Copyright 2005 American Chemical Society.

According to the X-ray crystal structure of ASR (Figure 4), it is similar to those of other archaeal-type rhodopsins. ASR accommodates both all-*trans* and 13-*cis* retinal in the ground state, which can be interconverted by illumination with blue (480 nm) or orange (590 nm) light (Figure 3) [16]. Such photochromic behavior has never been observed in other archaeal rhodopsins such as BR, HR, SRI and SRII, being characteristic to ASR. These results suggested that ASR could be a photochromic color sensor, whereas nothing was well-known about the structural changes and scheme of its photochromic reactions when we started the study.

**Figure 5.**
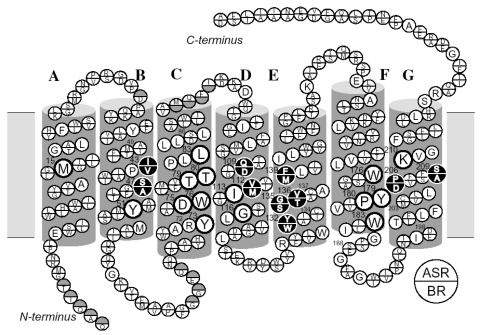
Comparison of amino acid sequences of ASR and BR. The transmembrane topology is based on the crystallographic three-dimensional structures. The sequence alignment was done using CLUSTAL W [21] with the default settings. Single letters in a circle denote residues common to ASR and BR. The residues that are different in ASR and BR are denoted at the top and bottom of the circles, respectively. The residues forming the retinal binding site within 5 Å of the chromophore are shown by bold or filled circles. This figure is reprinted with permission from Furutani *et al.* [20]. Copyright 2005 American Chemical Society.

Amino acid sequence comparison between ASR and BR reveals that some important residues for pumping protons are replaced in ASR. The proton donor to the Schiff base (Asp96 in BR) and one of proton release groups (Glu194 in BR) are replaced by serine residues, Ser86 and Ser188, respectively (Figure 5).

**Figure 6.**
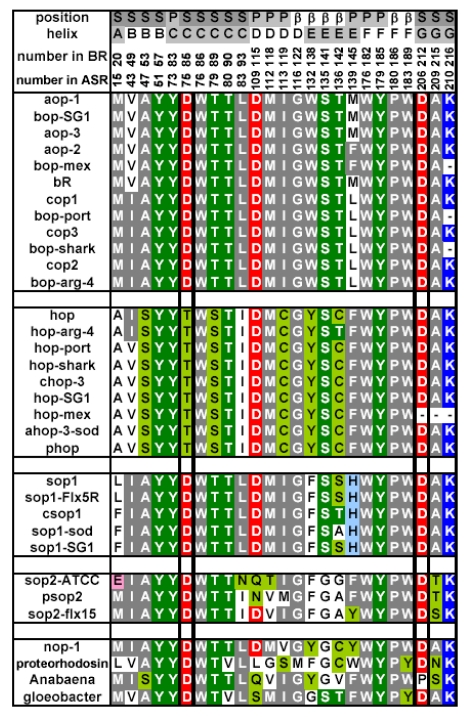
The 25 amino acid sequence of archaeal-type rhodopsin around retinal. First, second, third, fourth and fifth categories represent the families of bacteriorhodopsin, halorhodopsin, sensory rhodopsin I, sensory rhodopsin II and other archaeal-type rhodopsins, respectively. position S: near the Schiff base, P: near the polyene chain, β: around the β-ionon ring.

Ten amino acid residues out of twenty-five which constitute the retinal binding site are different from those of BR, probably accounting for the different absorption maximum and photochromic behavior of ASR. Among them, the most characteristic replacement is Pro206 located at the position of Asp212 in BR, which is one of the counterions of the Schiff base and a well conserved amino acid residue in archaeal type rhodopsins (Figure 6). The influence of Pro206 on the hydrogen bonds around the Schiff base should be studied precisely for elucidating the difference in the structural changes of retinal and protein between ASR and BR upon their activation.

As shown above, ASR is a unique archaeal-type rhodopsin. However, the molecular properties, particularly the nature of its photochromic behavior, were much less known. Thus, in the last five years, we have studied various properties of ASR, mainly by use of spectroscopic techniques. Since ASR possesses visible absorption, UV-visible spectroscopy is the basic technique to study the properties of this molecule. Low-temperature UV-visible spectroscopy was used to study the photochromism of ASR described in Section 4. In the photochromic reaction, the isomeric states of the retinal chromophore play an important role, and HPLC analysis is the direct method to determine the isomeric composition of the retinal chromophore. We used this method in the work described in Section 3. On the other hand, in our studies we have mostly used low-temperature Fourier-transform infrared (FTIR) spectroscopy [22,23]. Infrared frequencies cover the 4,000–100 cm^-1^ region, which corresponds to the molecular vibrations of interest, so infrared spectroscopy is a particularly suitable experimental tool to study structural changes in proteins. In this review article, Sections 2 and 3 cover the local structural analysis of retinal photoisomerization (77 K) of the all-*trans* and 13-*cis* forms of ASR, respectively, while Section 5 describes cytoplasmic surface structural perturbation of all-*trans* ASR at 170 K.

## 2. FTIR Spectroscopy of the All-*trans* Form of *Anabaena* Sensory Rhodopsin at 77 K: Hydrogen Bond of a Water between the Schiff Base and Asp75

As mentioned, comparison of the amino acid sequences of ASR and BR shows that some important residues for the proton pump in BR are replaced in ASR (Figure 5). The most characteristic replacement is Pro206 at the corresponding position of Asp212 in BR, Asp212 counterions a counterion complex of the Schiff base in BR, and the aspartate is highly conserved among archaeal rhodopsins. How is the hydrogen bonding network around the Schiff base modified in ASR by the presence of Pro206?

We have used low-temperature Fourier transform infrared (FTIR) spectroscopy to detect and study X-H and X-D (X = O, N) stretching vibrations in the mid-infrared region (4,000–1,800 cm^-1^) [22,23]. These vibrations are direct indications of the hydrogen bonding network, including internal water molecules. In fact, comparison of the K intermediate (BR_K_) minus BR difference spectra between hydration with D_2_O and D_2_^18^O in the X-D stretching region (2,700–1,800 cm^-1^) enabled us to assign the O-D stretching vibrations of water molecules not only with a weak hydrogen bond (at >2,500 cm^-1^) but also with a strong hydrogen bond (at <2,400 cm^-1^). A mutation study showed that one of the O-D stretches (2,171 cm^-1^) originates from a bridging water molecule between the Schiff base and its counterion (Asp85) [25]. Hydration switch of the water plays an important role in the proton transfer reaction in BR [26]. In addition and interestingly, comprehensive studies of BR mutants and other rhodopsins have revealed that strongly hydrogen-bonded water molecules are only found in the proteins exhibiting proton pump activities [27]. This suggests that a strongly hydrogen bonded water molecule that bridges the Schiff base and its counterion is essential for proton pumping, but in terms of this idea, our FTIR study of ASR is intriguing, because ASR possesses a bridged water like BR [16], but does not pump protons [15].

Here, we applied low-temperature FTIR spectroscopy to the all-*trans* form of ASR, and compared the difference spectra at 77 K with those of BR. The K intermediate minus ASR difference spectra show that the retinal isomerizes from the all-*trans* to the distorted 13-*cis* form, like BR. The N-D stretching of the Schiff base was observed at 2,163(−) and 2,125(−) cm^-1^, while the O-D stretchings of water molecules were observed in the region >2,500 cm^-1^. These results indicate that the protonated Schiff base forms a strong hydrogen bond with a water molecule, which is connected to Asp75 with a weak hydrogen bond. This result supports our working hypothesis about the strong correlation between the proton pump activity and the existence of strongly hydrogen bonded water molecules in archaeal rhodopsins. We shall discuss in some detail the structural reason why the bridged water molecule does not form a strong hydrogen bond in ASR.

In spectroscopic studies of archaeal rhodopsins, it is important to distinguish between the separate the photocyclization products of the all-*trans* from the 13-*cis* forms. In the case of BR, a well-known light adaptation procedure leads to a complete all-*trans* form. On the other hand, Vogeley *et al.* reported that ASR has a maximal amount of the all-*trans* form in the dark (>75%), while light adaptation rather decreases the amount of the all-*trans* form [16]. This was reproduced in this study, and hence, we used the dark-adapted ASR sample. The absorption maximum of the all-*trans* enriched ASR was located at 549 nm, which was the same value previously reported [16]. Low-temperature UV-visible spectroscopy of ASR showed that the red-shifted intermediate (ASR_K_) is formed at 77 K. The difference absorption maximum was located at 593 nm, and we estimated the absolute absorption maximum of ASR_K_ at 589 nm (data not shown).

### 2.1. Comparison of the Difference Infrared Spectra Obtained by the Photoreactions of Anabaena Sensory Rhodopsin (ASR) at 77 K with Those of Bacteriorhodopsin (BR)

**Figure 7.**
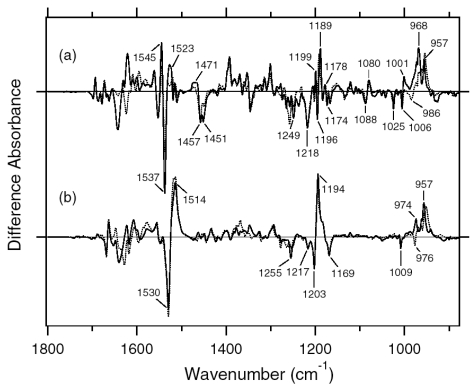
The ASR_K_ minus ASR (a) and the BR_K_ minus BR (b) spectra in the 1,800–850 cm^-1^ region measured at pH 7 and 77 K upon hydration with H_2_O (solid line) and D_2_O (dotted line), respectively. In the hydrated film, ASR molecules are oriented randomly, while BR molecules are highly oriented. Spectrum in (b) is reproduced from Kandori *et al.* [28], where the sample window is tilted by 53.5°. One division of the y-axis corresponds to 0.005 absorbance units. This figure is reprinted with permission from Furutani *et al.* [20]. Copyright 2005 American Chemical Society.

Figure 7 shows the ASR_K_ minus ASR (a) and BR_K_ minus BR spectra (b), which were measured at 77 K upon hydration with H_2_O (solid lines) and D_2_O (dotted lines). Unlike those of BR, the difference spectra of ASR contain a mixture of photoproducts of the all-*trans* and 13-*cis* form. However, we estimated by use of the marker band (1,178 cm^-1^) that the 13-*cis* form contribution under our illumination conditions is less than 20% (see below). Almost all vibrational bands described in this chapter originate from the photoreaction of the all-*trans* form.

The negative band at 1,537 cm^-1^ corresponds to the ethylenic stretching vibration of the all-*trans* chromophore in ASR, which exhibits an absorption maximum at 549 nm [16]. The frequency is in good agreement with the well-known linear correlation between the ethylenic stretching frequencies and absorption maxima for various retinal proteins [29]. In the case of BR, the bands at 1,530(−)/1,514 (+) cm^-1^ correspond to the ethylenic stretching vibrations of the unphotolyzed and K intermediate (BR_K_) states, respectively (Figure 7b). On the other hand, two positive bands appeared at 1,545 and 1,523 cm^-1^ for ASR (Figure 7a). According to the ethylenic stretching frequencies and absorption maxima correlation we predicted the ethylenic stretch of ASR_K_ (589 nm) to be at 1,525 cm^-1^. Therefore, the 1,523 cm^-1^ band is likely to be the latter, and the band at 1,545 cm^-1^ can possibly be assigned to the amide II mode. A similar observation was made for halorhodopsin [30], where the K intermediate exhibits two positive bands at 1,538 and 1,514 cm^-1^ with a negative band at 1,525 cm^-1^.

Remarkable spectral differences between ASR and BR were seen in the 1,500–1,450 cm^-1^ region. Two negative bands at 1,457 and 1,451 cm^-1^ and a positive band at 1,471 cm^-1^ were observed for ASR (Figure 7a). Among these three bands, the 1,457 cm^-1^ band is insensitive to H-D exchange, whereas the bands at 1,471 and 1,451 cm^-1^ are reduced to the half the intensity in D_2_O. On the other hand, such strong bands are absent for BR (Figure 7b). This frequency region corresponds to the imide II vibrations of proline.

### 2.2. Comparison of the Vibrational Bands of the Retinal Chromophore between ASR and BR

**Figure 8.**
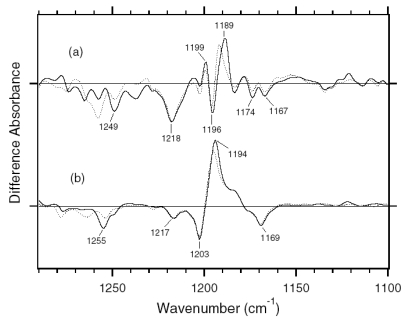
The ASR_K_ minus ASR (a) and the BR_K_ minus BR (b) spectra in the 1,290–1,100 cm^-1^ region, which correspond to C-C stretching vibrations and N-H in-plane bending vibrations of the retinal chromophore. The sample was hydrated with H_2_O (solid lines) or D_2_O (dotted lines). One division of the y-axis corresponds to 0.004 absorbance units. This figure is reprinted with permission from Furutani *et al.* [20]. Copyright 2005 American Chemical Society.

The C-C stretching vibrations of retinal in the 1,290–1,100 cm^-1^ region are sensitive to the local structure of the chromophore. In Figure 8b, the negative bands at 1,217, 1,169, 1,254, and 1,203 cm^-1^ were assigned to the C8-C9, C10-C11, C12-C13, and C14-C15 stretching vibrations of BR, respectively [31]. These frequencies are characteristic of the all-*trans* retinal protonated Schiff base, though the frequencies are higher because of the charge delocalization of the retinal molecule in BR. Upon formation of BR_K_, retinal isomerizes to the 13-*cis* form, resulting in the appearance of a strong positive band at 1,194 cm^-1^, which is assigned to C10-C11 and C14-C15 stretching vibrations [32]. A more complex spectral feature was observed for ASR in the 1,290-1,100 cm^-1^ region (Figure 8a). One reason is that the photoreaction of the 13-*cis* form to its photoproduct contributes to these spectra. It is known that a positive band at ∼1,180 cm^-1^ is a marker band of such a reaction in BR [33]. Similarly, in this study for ASR, we found that the bands at 1,183(−)/1,178(+) cm^-1^ increase in intensity when illumination wavelengths are changed. Thus, we interpreted that these bands originate from the photoreaction of the 13-*cis* form in ASR as well as in BR. In other words, we established the illumination conditions to maximally reduce the bands at 1,183(-)/1,178(+) cm^-1^ in this study.

In the case of the all-*trans* form of ASR, the negative bands at 1,218, 1,174 (and/or 1,167), 1,249, and 1,196 cm^-1^ were tentatively assigned to the C8-C9, C10-C11, C12-C13, and C14-C15 stretching vibrations, respectively (Figure 8a). These frequencies are similar to those of BR (each frequency difference is <10 cm^-1^), supporting the fact that the retinal configuration of ASR in the dark-adapted state is all-*trans*. However, the relatively large difference in C12-C13 (6 cm^-1^) and C14-C15 (7 cm^-1^) stretching vibrations suggests that the retinal structure near the Schiff base region is somehow different in ASR and BR. In addition, the intensity of the band at 1,218 cm^-1^ is three times larger than that of BR, which also suggests different retinal structure around the C8-C9 bond. Upon formation of ASR_K_, the retinal molecule is considered to isomerize to the 13-*cis* form in analogy to the case of BR. However, unlike BR, there are three positive bands at 1,199, 1,189, and 1,149 cm^-1^. The 1,199 cm^-1^ band is not sensitive to H-D exchange, suggesting the origin as a C-C stretching vibration in the polyene chain of the retinal molecule. The bands at 1,189 and 1,149 cm^-1^ are upshifted upon hydration with D_2_O, suggesting that they are influenced by the Schiff base vibration. The 1,189 cm^-1^ band can be assigned to the C14-C15 stretching vibration, while the 1,149 cm^-1^ band is difficult to identify at present. The downshift of the C14-C15 stretching vibration from 1,196 to 1,189 cm^-1^ upon formation of ASR_K_ suggests that the retinal configuration is 13-*cis* in ASR_K_. Splitting into two negative bands at 1,174 and 1,167 cm^-1^ may suggest the presence of a positive band at 1,171 cm^-1^, which can be assigned to the C10-C11 stretching vibration.

The H-D exchangeable band at 1,255 cm^-1^ was assigned to one of the modes containing the N-H in-plane bending vibration of the Schiff base of BR [34], while similar negative bands appear at 1,249 cm^-1^ in the spectra of ASR. The band disappearing upon hydration with D_2_O can be assigned to the modes of the Schiff base. The neighboring negative band at 1,237 cm^-1^ is also sensitive to deuteration and seen only in ASR, but its origin remains unknown. The result suggests that the hydrogen bonding environment of the Schiff base of ASR is similar to that of BR.

**Figure 9.**
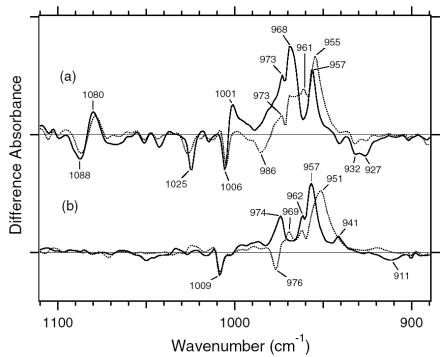
The ASR_K_ minus ASR (a) and the BR_K_ minus BR (b) spectra in the 1,110–890 cm^-1^ region, which correspond to hydrogen-out-of-plane (HOOP) vibrations of the retinal chromophore. The sample was hydrated with H_2_O (solid lines) or D_2_O (dotted lines). One division of the y-axis corresponds to 0.002 absorbance units. This figure is reprinted with permission from Furutani *et al.* [20]. Copyright 2005 American Chemical Society.

The difference spectra in the 1,110–890 cm^-1^ region are expanded in Figure 9. Hydrogen-out-of-plane (HOOP), N-D in-plane bending, and methyl rocking vibrations are observed here, and the presence of strong HOOP modes represents the distortion of the retinal molecule at the corresponding position. The most intense HOOP band in the BR_K_ minus BR difference spectra (Figure 9b) was observed at 957 cm^-1^ (in H_2_O) and 951 cm^-1^ (in D_2_O), which were assigned to the C15-H HOOP vibration of BR_K_ [34]. The origins of the bands at 941, 962, and 974 cm^-1^ remain unknown, but they may be assigned to HOOP vibrations. On the other hand, the weak negative band at 911 cm^-1^ was assigned to the C15-H and N-H HOOP vibrations of the original state of BR [35]. These results have been interpreted as an increase in the retinal distortion around the Schiff base upon the retinal isomerization in BR. In the case of ASR, similar but slightly upshifted bands were observed. The positive bands at 1,001, 973, 968, and 957 cm^-1^ of ASR_K_ (Figure 9a) possibly correspond to those at 974, 962, 957, and 941 cm^-1^ of BR_K_, respectively (Figure 9b). The negative bands at 932 and 927 cm^-1^ have probably the same origin as that at 911 cm^-1^ in BR, which was assigned to the C15-H and N-H HOOP vibrations [35].

The negative band at 976 cm^-1^ and the positive band at 969 cm^-1^ in Figure 9b were assigned to the N-D in-plane bending vibrations of BR and BR_K_, respectively [34]. The 1,009 cm^-1^ band is insensitive to H-D exchange and was assigned to the methyl rocking vibration of the retinal in BR. The band at 1,006 cm^-1^ in Figure 9a can also be assigned to the methyl rocking vibration in ASR similarly. On the other hand, the bands at 1,088(−), 1,080(+), and 1,025(−) cm^-1^ are highly characteristic of the ASR_K_ minus ASR difference spectra, and never observed in other archaeal-type rhodopsins such as BR, *p*pR, and NR [34,36,37]. According to the literature, the antisymmetric NC_3_ stretchings of tertiary amines appear in the 1,250−1,000 cm^-1^ region [38]. Thus, these bands may originate from the skeletal vibrations of Pro206 as well as those at 1,471(+), 1,457(−), and 1,451(−) cm^-1^ (Figure 7a).

**Figure 10.**
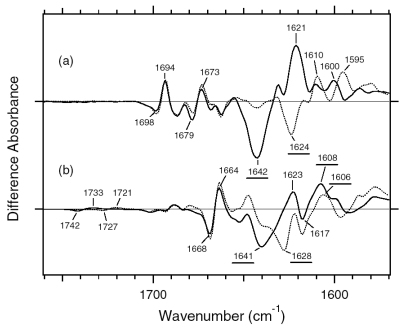
The ASR_K_ minus ASR (a) and the BR_K_ minus BR (b) spectra in the 1,760–1,570 cm^-1^ region, most of which are ascribable for vibrations of the protein moiety. The underlined peaks are C=N stretching vibrations of the chromophore. The sample was hydrated with H_2_O (solid lines) or D_2_O (dotted lines). One division of the y-axis corresponds to 0.003 absorbance units. This figure is reprinted with permission from Furutani *et al.* [20]. Copyright 2005 American Chemical Society.

C=N stretching vibrations of the protonated retinal Schiff base are observed in the 1,650–1,600 cm^-1^ region (Figure 10). The C=N stretching vibrations are sensitive to H-D exchange, and the difference in frequency has been considered as the probe for its hydrogen bonding strength [39,40], that is, the larger the difference is, the stronger the hydrogen bond is. The C=NH and C=ND stretching vibrations of BR were observed at 1,641 and 1,628 cm^-1^, while those of BR_K_ were at 1,608 and 1,606 cm^-1^, respectively [41]. The former difference in frequency is 13 cm^-1^, and the latter is 2 cm^-1^, suggesting that the protonated Schiff base forms a hydrogen bond in BR that is broken upon retinal isomerization. The C=N stretches were observed at 1,642 (C=NH) and 1,624 cm^-1^ (C=ND) in ASR, and its difference is 18 cm^-1^, suggesting that the hydrogen bonding strength is stronger than that of BR. On the other hand, it is difficult to assign the positive bands because of the more complicated spectral features. There are two sets of candidates for the C=N stretching vibrations of ASR_K_. One set is the bands at 1,621 (C=NH) and 1,610 cm^-1^ (C=ND), while another set is the bands at 1,600 (C=NH) and 1,595 cm^-1^ (C=ND). The differences in frequency are 11 and 5 cm^-1^ for the former and latter, respectively. If the former is the case, the hydrogen bond may not be broken upon retinal isomerization in ASR. Conclusive assignment of the C=N stretching of ASR_K_ needs stable isotope labeling on the Schiff base, which is shown in next chapter. On the other hand, the N-D stretching vibration of the Schiff base in D_2_O provides more direct information about the hydrogen bond of the Schiff base, as described below.

### 2.3. Comparison of the C=O Stretching Vibrations of Carboxylate, Carbonyl, and Amide Groups of the Protein Moiety between ASR and BR

In the BR_K_ minus BR difference spectra (Figure 10b), the bands at 1,742 and 1,733 cm^-1^ were assigned to the C=O stretching vibrations of the protonated Asp115, which are downshifted upon hydration with D_2_O [42]. In contrast, there is no band in the same frequency region of the ASR spectra (Figure 10a), implying that Asp and Glu residues are located far from the retinal molecule even if they are protonated. ASR has a glutamine residue at the corresponding position of Asp115 in BR, whose vibrational bands are probably observed at 1,698(−) and 1,694(+) cm^-1^ (Figure 10a). Similar bands were also observed at 1,704(−) and 1,700(+) cm^-1^ in the difference spectra of *p*pR, which has an asparagine residue at the corresponding position [43]. These observations suggest that the structural changes around Asp115 in BR are similar among ASR, BR, and *p*pR.

The band pairs at 1,668(−)/1,664(+) cm^-1^ and at 1,623(+)/1,617(−) cm^-1^ were assigned to the amide I C=O stretching vibrations. The former was assigned to the amide I of α_II_ helix [44] and the latter to the amide I of Val49 [45]. In the case of ASR, a band pair at 1,679(−)/1,673(+) cm^-1^ could be similar in origin to the bands at 1,668(−)/1,664(+) cm^-1^ in BR. It should be noted that the spectral changes of amide I vibrations at <1,660 cm^-1^ are much smaller in ASR than in BR, which is clearly seen in D_2_O. This suggests that the structural changes of the peptide backbone in ASR upon retinal isomerization are very small. On the other hand, the structural perturbation of Pro206 was suggested for ASR.

### 2.4. Comparison of the X-D Stretching Vibrations between ASR and BR

**Figure 11.**
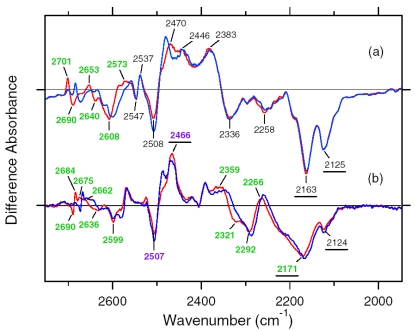
Comparison of the difference infrared spectra of ASR (a) and BR (b) hydrated with D_2_O (red lines) or D_2_^18^O (blue lines) in the 2,730-1,950 cm^-1^ region. Green-labeled frequencies correspond to those identified as water stretching vibrations. Purple-labeled frequencies are O-D stretches of Thr89 [48,49], while the underlined frequencies are N-D stretches of the Schiff base [41]. Spectrum in (b) is reproduced from Tanimoto *et al.* [26], where the sample window is tilted by 53.5°. One division of the y-axis corresponds to 0.0007 absorbance units. This figure is reprinted with permission from Furutani *et al.* [20]. Copyright 2005 American Chemical Society.

X-D stretching vibrations of protein and water molecules appear in the 2,750–1,950 cm^-1^ region (Figure 11). A spectral comparison between the samples hydrated with D_2_O and D_2_^18^O identifies O-D stretching vibrations of water molecules which change their frequencies upon retinal photoisomerization. Green-labeled bands in Figure 11 can be assigned to the O-D stretching vibrations of water because of the isotope shift. In BR, six negative peaks at 2,690, 2,636, 2,599, 2,321, 2,292, and 2,171 cm^-1^ were earlier assigned to vibrations of water molecules (Figure 11b) [26,46]. The bands are widely distributed over the possible frequency range for stretching vibrations of water. Since the frequencies of the negative peaks at 2,321, 2,292, and 2,171 cm^-1^ are much lower than those of fully hydrated tetrahedral water molecules [46], the hydrogen bonds of those water molecules must be very strong, possibly indicating their association with negative charges. Indeed, we assigned the 2,171 cm^-1^ band to the O-D group of a water molecule associated with deprotonated Asp85 [25]. This water molecule, called water 402 in the crystal structure of BR (PDB entry 1C3W), is located between the Schiff base and Asp85 (Figure 4). A previous QM/MM calculation of the Schiff base region of BR also supported the existence of an extremely strong hydrogen bond between water 402 and Asp85 [47]. Water stretching vibrations of BR_K_ tend to be higher in frequency, implying that the overall hydrogen bonding becomes weaker upon photoisomerization.

In contrast, interestingly only three negative peaks at 2,690, 2,640, and 2,608 cm^-1^ could be assigned to the O-D stretching vibrations of water in ASR (Figure 11a). The bands at 2,701, 2,653, and 2,573 cm^-1^ were assigned as water stretching vibrations of ASR_K_. It should be emphasized that there are no water bands in the <2,400 cm^-1^ region, which is a significant difference from the published results for BR and *p*pR. In the case of *p*pR, two pairs of peaks were observed in the <2,400 cm^-1^ region, located at 2,369(+)/2,307(−) cm^-1^ and at 2,274(+)/2,215(−) cm^-1^ [47]. Since ASR has a bridged water molecule between the Schiff base and Asp75 (Figure 4) as well as BR and *p*pR, one may expect similar water bands at <2,400 cm^-1^. However, that is not the case for ASR. We will discuss the structural reason for the lack of strongly hydrogen bonded water molecules below.

The frequency region shown in Figure 11 also contains X-D stretching vibrations other than water molecules. In the BR_K_ minus BR spectrum, the bands at 2,507(−)/2,466(+) cm^-1^ labeled in purple and the underlined bands at 2,466(+), 2,171(−), and 2,124(−) cm^-1^ were assigned to the O-D stretching vibrations of Thr89 [48,49] (the corresponding residue in ASR is Thr79) and the N-D stretching vibrations of the retinal Schiff base [41], respectively. Thus, the negative 2,171 cm^-1^ band contains both the O-D stretch of water and the N-D stretch of the Schiff base. In the ASR_K_ minus ASR spectrum, there are 10 bands other than water bands: 2,547(−), 2,537(+), 2,508(−), 2,470(−), 2,446(+), 2,383(+), 2,336(−), 2,258(−), 2,163(−), and 2,125(−) cm^-1^. The bands at 2,547(−)/2,537(+) cm^-1^ are attributed to the H-D unexchangeable S-H stretching vibration of a cysteine residue as described below. The bands at 2,508(−)/2,470(+) cm^-1^ can be assigned to the O-D stretching vibrations of Thr79 in analogy to BR. The O-D frequencies of Thr79 in ASR and ASR_K_ (2,508 and 2,470 cm^-1^) are almost identical to those of Thr89 in BR and BR_K_ (2,507 and 2,466 cm^-1^), respectively, indicating that the strength of hydrogen bonding between Thr79 and Asp75 is identical to that between Thr89 and Asp85 in BR.

Though not assigned directly by use of the labeled protein, the bands at 2,163 and 2,125 cm^-1^ are likely to originate from N-D stretching of the Schiff base, whose frequencies are very similar to those in BR (2,171 and 2,124 cm^-1^). This fact indicates similar hydrogen bonding strengths between ASR and BR. The slightly lower frequency of the strong band (2,163 cm^-1^ in ASR and 2,171 cm^-1^ in BR) may correspond to the results obtained for the C=N stretching vibrations as shown before (Figure 10). The analysis of the C=N stretching vibrations of ASR_K_ suggested two possibilities for the hydrogen bonding strength of the Schiff base. Figure 10a clearly shows the presence of the negative bands at 2,163 and 2,125 cm^-1^, implying that the N-D stretch is upshifted in ASR_K_. We infer that one of the bands at 2,470, 2,446, and 2,383 cm^-1^ can be assigned to the N-D stretch in ASR_K_. Thus, we can safely conclude that the hydrogen bond of the Schiff base in ASR becomes much weaker upon retinal photoisomerization as well as in BR.

### 2.5. S-H Stretching Vibrations of the Cysteine Residues

**Figure 12.**
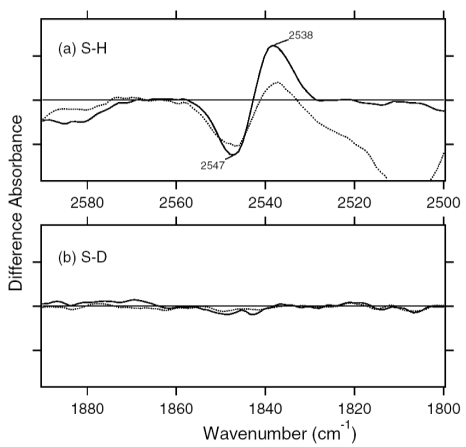
The ASR_K_ minus ASR spectra in the 2,590–2,500 cm^-1^ (the upper panel) and 1,890-1,800 cm^-1^ (the lower panel) region, which correspond to S-H and S-D stretching vibrations of cysteine residues, respectively. The sample was hydrated with H_2_O (solid lines) or D_2_O (dotted lines). One division of the y-axis corresponds to 0.0001 absorbance units. This figure is reprinted with permission from Furutani *et al.* [20]. Copyright 2005 American Chemical Society.

Figure 12 shows the ASR_K_ minus ASR spectra in the 2,590–2,500 cm^-1^ (top panel) and 1,890–1,800 cm^-1^ (bottom panel) regions, which correspond to S-H and S-D stretching vibrations of cysteine residues, respectively. There are a negative band at 2,547 cm^-1^ and a positive band at 2,538 cm^-1^, while no band is observed in the S-D stretching upon hydration with D_2_O.

In fact, S-H stretching vibrations in D_2_O are observed in Figure 11a (2,547 and 2,537 cm^-1^). The S-H stretching frequency of cysteine appears in the 2,580–2,525 cm^-1^ region. Thus, the frequency change from 2,547 to 2,538 cm^-1^ suggests that the cysteine forms a considerably strong hydrogen bond upon retinal isomerization. The non H-D-exchangeable nature of the cysteine S-H group presumably results from either the hydrophobic environment or the strong hydrogen bond.

The lower-frequency shift in ASR is the opposite of the cysteine signal in the NR_K_ minus NR spectra [37]. In addition, the H-D exchange is different between ASR and NR. These facts suggest that the cysteine residues are located in different environments and their hydrogen bonds change differently. There are three cysteine residues in ASR, Cys134 and Cys137 in helix E and Cys203 in helix G. Not all of them are conserved in archaeal-type rhodopsin, but Cys134 and Cys137 are located at a position similar to that of Cys170 in NR, which is conserved in halorhodopsin. The X-ray crystal structure of ASR also revealed that only the S-H group of Cys203 is directed to the inside of the protein. From these results, the observed band can be assigned to the S-H stretching of Cys203.

### 2.6. Unique Structure of the All-trans Form of ASR

In this study, we measured the ASR_K_ minus ASR spectra by means of low-temperature FTIR spectroscopy. For this purpose, ASR was expressed in *E. coli*, and the wild-type protein was reconstituted into PC liposomes. It is noted that the ASR molecule is not embedded in the native membrane, which could modify the FTIR spectra. For instance, H-D exchange could be different between PC liposomes and the native membrane, a fact that should be elucidated in the future. However, this study focuses on the structural changes near the retinal upon photoisomerization, and the light-induced difference FTIR spectra are not significantly affected by different lipid environments.

Despite the presence of the 13-*cis* form, the obtained spectra are predominantly due to the photoreaction of the all-*trans* form, and the spectra were compared with those of BR. These results clearly show that the all-*trans* to 13-*cis* photoisomerization takes place in ASR like in BR, though the C-C stretching and HOOP vibrations are somehow different. The protonated Schiff base forms a strong hydrogen bond in ASR, presumably with the bridged water (Figure 4), and the hydrogen bond is cleaved by the rotation of the N-H (N-D) group, as in BR. We also observed S-H stretches of a cysteine residue which is insensitive to hydration with D_2_O. We observed small amide I bands and large bands that can be ascribed to imide II [1,471(+), 1,457(−), and 1,451(−) cm^-1^] and NC_3_ [1,088(−) and 1,080(+) cm^-1^] stretchings of proline residues. A previous resonance Raman spectroscopic study showed that the imide II vibration of the X-Pro bond appears at around 1,450 cm^-1^ [50]. BR has three Pro residues in the transmembrane region, Pro50, Pro91, and Pro186 (Figure 5). The previous FTIR study suggested that the environment around these proline residues changes upon retinal isomerization via observation of the isotope effect of [^15^N]proline in the 1,450–1,420 cm^-1^ region [51]. It should be noted that spectral changes are much smaller in BR than in ASR in this frequency region. In the case of ASR, there are additional three Pro residues (Figure 5). It is Pro206, a corresponding residue of Asp212 in BR (Figure 4). Figure 4 shows that the peptide C=O group of Pro206 forms a hydrogen bond with the peptide amide (N-H group) of Lys210, which connects to a retinal chromophore. Thus, retinal photoisomerization strongly perturbs the peptide C-N bond of Pro206 in ASR, presumably leading to the appearance of these unusually intense bands in the 1,500–1,450 cm^-1^ region. It should be noted, however, that we can conclude this argument only when these bands are assigned by use of [^15^N] proline-labeled ASR.

A significant difference between ASR and BR was seen for water bands. We have so far observed the O-D stretching vibrations of water molecules under strongly hydrogen bonded conditions in the BR_K_ minus BR and *p*pR_K_ minus *p*pR difference spectra [26,36,46]. The X-ray crystal structures of BR and *p*pR reported the presence of a bridged water molecule between the Schiff base and its counterion (Asp85 in BR and Asp75 in *p*pR) [7,52,53]. Therefore, the hydrogen bond of the water is expected to be strong, and such strongly hydrogen bonded water molecules were observed in the FTIR studies. The water molecules possess O-D stretches at 2,400–2,100 cm^-1^ in D_2_O [23,27]. Since ASR has a bridged water molecule between the Schiff base and Asp75 (Figure 4) as well as BR and *p*pR, one may expect similar water bands at <2,400 cm^-1^. However, that was not the case for ASR. Therefore, the structural reason for the lack of strongly hydrogen bonded water molecules has to be explained on the basis of the structural background. Since both structures of ASR and BR are known (Figure 4), we will try to explain the reason here.

**Figure 13.**
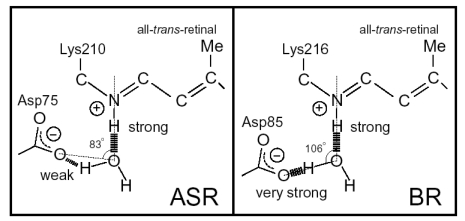
Schematic drawing of hydrogen bonds of the water molecule locating between the protonated Schiff base and its counterion. A part of all-*trans* retinal is depicted, β-ionon ring and ethylenic part from C6 to C12 are omitted. The numbers are the angle of the N-O-O atoms derived from the crystal structures of ASR and BR (PDB entries are 1XIO and 1C3W, respectively). Hydrogen bonds are indicated by the dashed lines with their strength. This figure is reprinted with permission from Furutani *et al.* [20]. Copyright 2005 American Chemical Society.

Our analysis of the Schiff base mode (C=N stretch and N-D stretch) in ASR showed that the hydrogen bonding strength of the Schiff base is similar in ASR and BR. This observation is consistent with the similar distance between the Schiff base nitrogen and water oxygen (2.6 Å for ASR and 2.9 Å for BR). A slightly stronger hydrogen bond in ASR than in BR is also consistent with the distance that is shorter in ASR. In contrast, water bands in ASR were entirely different from those in BR, although the distance between the water oxygen and the oxygen of the counterion are similar (2.7 Å for ASR and 2.6 Å for BR). The O-D stretch of the bridged water in BR is located at 2,171 cm^-1^ (Figure 11b), whereas that in ASR is probably one of the bands at 2,690, 2,640, and 2,608 cm^-1^ (Figure 11a). How is such difference observed between ASR and BR? It may be explained by the difference in the geometry of the hydrogen bond. Figure 13 shows that the N-O_water_-O_Asp75_ (the Schiff base nitrogen, the water oxygen, and the oxygen of Asp75, respectively) angle in ASR is 83°. The corresponding N-O_water_-O_Asp85_ angle in BR is 106° (Figure 13). As the consequence, if the water oxygen fully accepts the hydrogen bond of the Schiff base, the O-H group of water points toward the oxygen of Asp85 in BR, but not toward that of Asp75 in ASR (Figure 13). Such a small difference in angle possibly determines the hydrogen bonding strength of water molecules.

On the basis of our FTIR studies of BR mutants and other rhodopsins, we have found an interesting correlation between strongly hydrogen bonded water molecules and proton pump activity. Among various BR mutant proteins we have studied, only D85N and D212N lack strongly hydrogen bonded water molecules. Other BR mutants possess their O-D stretches at <2,400 cm^-1^, which include T46V, R82Q, R82Q/D212N, T89A, D96N, D115N, Y185F, and E204Q [54]. Among these mutants, only D85N and D212N do not pump protons. Therefore, strongly hydrogen bonded water molecules are only found in the proteins exhibiting proton pumping activities. The correlation between proton pumping activity and strongly hydrogen bonded water molecules is true not only for BR mutants but also for various rhodopsins. Whether rhodopsins possess strongly hydrogen bonded water molecules has been examined systematically. We found that BR and *pharaonis* phoborhodopsin [26,36,46], both of which pump protons, possess such water molecules (O-D stretch at <2,400 cm^-1^ in D_2_O). In contrast, strongly hydrogen bonded water molecules were not observed for halorhodopsin [55], *Neurospora* rhodopsin [37], and bovine rhodopsin [56]. It is known that none of them pumps protons. Such comprehensive studies of archaeal and visual rhodopsins have thus revealed that strongly hydrogen bonded water molecules are only found in the proteins exhibiting proton pumping activities. Taken together, these results for ASR suggest that the strong hydrogen bonds of water molecules and their transient weakening may be essential for the proton pumping function of rhodopsins.

## 3. FTIR Study of the Photoisomerization Processes in the 13-*cis* and All-*trans* Forms of *Anabaena* Sensory Rhodopsin at 77 K

We then extended the low-temperature spectroscopic study at 77 K to the 13-*cis*, 15-*syn* form of ASR (13C-ASR). HPLC analysis revealed that light-adapted ASR with >560 nm light at 4 °C possesses 78% 13C-ASR, while dark-adapted ASR has AT-ASR predominantly (97%). Then, we established the illumination conditions to measure the difference spectra between 13C-ASR and its K state without subtracting the difference between AT-ASR and its K state. Spectral comparison between 13C-ASR and AT-ASR provided useful information on structure and structural changes upon retinal photoisomerization in ASR. In particular, previous X-ray crystallographic study of ASR reported the same protein structure for 13C-ASR and AT-ASR [16], whereas the present FTIR study revealed that protein structural changes upon retinal photoisomerization were significantly different between 13C-ASR and AT-ASR. The differences were seen for HOOP modes of the retinal chromophore, amide I, cysteine S-H stretch, the Schiff base N-D stretch, and water O-D stretch modes. These must trigger different global protein structural changes in each photoreaction cycle leading to the observed photochromic behavior.

**Figure 14.**
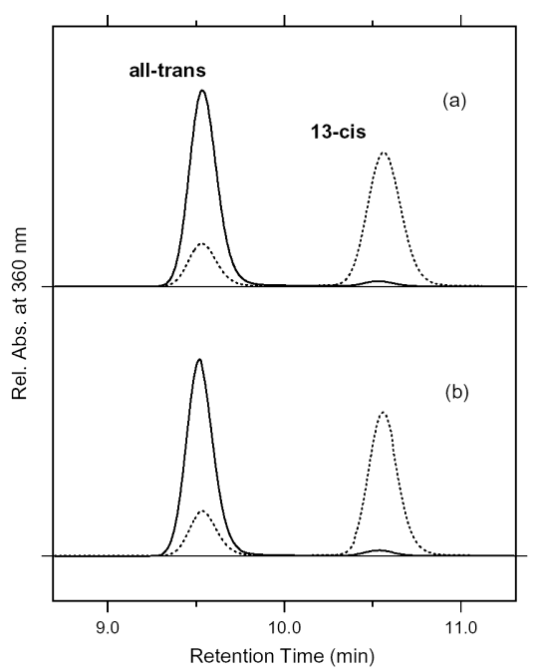
HPLC of chromophores extracted from ASR in DM micelles (a) and in PC liposomes (b). The detection beam was set at 360 nm. After the extraction, retinal oxime exists in 15-*syn* and 15-*anti* form. In the shown range of retention times, only the 15-*syn* form appears. We used area of both 15-*syn* and 15-*anti* forms for calculation of isomeric ratios. Dark-adapted ASR (solid lines) is in the all-*trans* form predominantly [AT-ASR; 95.5 ± 0.8% in (a) and 97.1 ± 0.1% in (b)], while light-adapted ASR (dotted lines) possesses more of the 13-*cis* form [13C-ASR; 78.1 ± 1.2% in (a) and 77.9 ± 1.7% in (b)]. This figure is reprinted with permission from Kawanabe *et al.* [59]. Copyright 2006 American Chemical Society.

Dark-adapted ASR is predominantly in the all-*trans* form, while the light adaptation process increases concentration of the 13-*cis* form [16,57]. This is in contrast to the case of BR, where light adaptation leads to a complete conversion into the all-*trans* form [58]. In this study, we attempted to establish the illumination conditions to accumulate the 13-*cis* form for DM-solubilized and PC-liposome-based ASR samples, using HPLC column chromatography. Panels a and b of Figure 14 show that the dark-adapted ASR (solid lines) possesses 95.5% and 97.1% all-*trans* form for the DM-solubilized and PC-liposome-based samples, respectively. On the other hand, illumination of ASR with >560 nm light for 1 min at 4 °C results in accumulation of 13C-ASR. HPLC analysis showed that the light-adapted ASR possesses 78.1% and 77.9% of the 13-*cis* form for the DM-solubilized and PC-liposome-based samples, respectively. Thus, the isomeric composition was not influenced by the reconstitution. Dark adaptation was a slow process, with half-time >1 h at 4 °C (data not shown). It should be noted that Sineshchekov *et al.* estimated the ratio of all-*trans* and 13-*cis* form to be 67:33 in the dark-adapted ASR and 20:80 in the light-adapted ASR [57]. The different value in the dark-adapted state may originate from the lipids for reconstitution (*E.coli* membrane in [57]).

**Figure 15.**
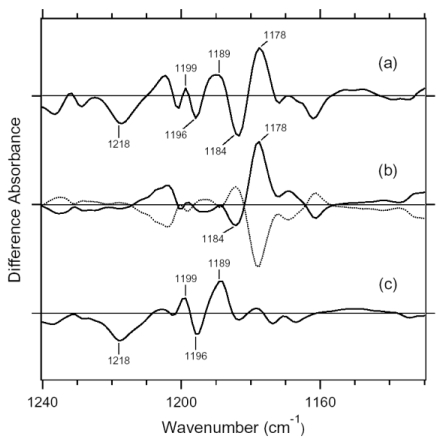
Difference FTIR spectra in the 1,240–1,130 cm^-1^ region measured at 77 K (in H_2_O), where the spectra before illumination were subtracted from those after illumination. Light-adapted ASR that contains 13C-ASR (78%) and AT-ASR (22%) was first illuminated with 501 nm light for 1 min (a). Then, illumination at >560 nm for 1 min converted a part of the photoproducts in (a) to the original state (dotted line in b). Subsequent illumination with 501 nm light yields the difference spectrum (solid line in b), which is a mirror image of the dotted spectrum. Repeated illuminations at >560 nm and at 501 nm yield the identical spectra. (c) The AT-ASR_K_ minus AT-ASR spectra are reproduced from Furutani *et al.* [20]. This figure is reprinted with permission from Kawanabe *et al.* [59]. Copyright 2006 American Chemical Society.

A hydrated film of ASR in PC liposomes was light-adapted as described above and then cooled to 77 K, followed by illumination at 501 nm. Figure 15a shows the light minus dark difference FTIR spectra of the light-adapted ASR. Vibrational bands at 1,218(−), 1,199(+), 1,196(−), and 1,189(+) cm^-1^ also appear in the AT-ASR_K_ minus AT-ASR (Figure 15c) [20], indicating that the conversion of AT-ASR to AT-ASR_K_ is included in the spectrum of Figure 15a. On the other hand, Figure 15a possesses additional strong peaks at 1,184 (−) and 1,178 (+) cm^-1^, suggesting the involvement of the photoreaction of 13C-ASR. In the previous study for AT-ASR, we illuminated AT-ASR_K_ at >590 nm for the photoreversion to AT-ASR [20]. In the present study, subsequent illuminations at >560 and 501 nm yielded the spectra shown in Figure 15b (dotted and solid lines, respectively). Lack of the bands at 1,218, 1,199, 1,196, and 1,189 cm^-1^ strongly suggests that the spectra should not contain the photoreaction of AT-ASR. In other words, the solid line in Figure 15b corresponds to the 13C-ASR_K_ minus 13C-ASR spectrum. In fact, the spectrum of Figure 15a is well constructed from the solid lines in Figure 15b,c (data not shown). In this way, we obtained the 13C-ASR_K_ minus 13C-ASR difference FTIR spectra without any subtraction of spectral contribution from AT-ASR.

It is likely that the photoequilibrium between AT-ASR and AT-ASR_K_ is not changed between illuminations at 501 nm and at >560 nm, so that further illumination with 501 nm and >560 nm yielded the difference spectra between 13C-ASR and 13C-ASR_K_. In this way, we obtained the 13C-ASR_K_ minus 13C-ASR difference FTIR spectra without any subtraction of spectral contribution from AT-ASR.

### 3.1. Comparison of the Difference Infrared Spectra of the Photoreactions of 13C-ASR and AT-ASR at 77 K in the 1,770–870 cm^-1^ Region

**Figure 16.**
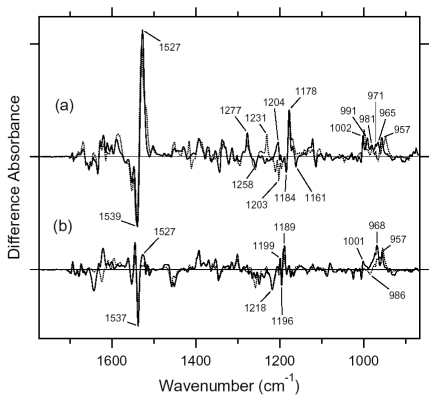
The 13C-ASR_K_ minus 13C-ASR (a) and the AT-ASR_K_ minus AT-ASR (b) spectra (pH 7) in the 1,800–800 cm^-1^ region measured at 77 K upon hydration with H_2_O (solid line) and D_2_O (dotted line), respectively. The spectra in panel b are reproduced from Furutani *et al.* [20]. One division of the y-axis corresponds to 0.007 absorbance units. This figure is reprinted with permission from Kawanabe *et al.* [59]. Copyright 2006 American Chemical Society.

Figure 16 shows the 13C-ASR_K_ minus 13C-ASR (a) and the AT-ASR_K_ minus AT-ASR spectra (b), which were measured at 77 K upon hydration with H_2_O (solid lines) and D_2_O (dotted lines). In Figure 16a, the negative band at 1,539 cm^-1^ corresponds to the ethylenic stretching vibration of the 13-*cis* chromophore in ASR, which exhibits the absorption maximum at 537 nm [16]. The frequency is in good agreement with the well-known linear correlation between the ethylenic stretching frequencies and absorption maxima for various retinal proteins [29]. Illumination results in the spectral downshift to 1,527 cm^-1^, indicating formation of the red-shifted K intermediate (13C-ASR_K_).

C-C stretching vibrations of the retinal in the 1,300–1,150 cm^-1^ region are sensitive to the local structure of the chromophore. In the 13C-ASR_K_ minus 13C-ASR spectrum in H_2_O, peaks are observed at 1,277(+), 1,258(−), 1,204(+), 1,184(−), 1,178(+), and 1,161(−) cm^-1^ (Figure 16a, solid line).

In the case of the 13-*cis* form of BR, appearance of a peak pair at 1,185(−) and 1,177(+) cm^-1^ was regarded as a marker of the formation of the all-*trans* photoproduct [33]. Similar bands at 1,184(−) and 1,178(+) cm^-1^ for 13C-ASR strongly suggest that 13C-ASR_K_ possesses the all-*trans* chromophore produced by photoisomerization of the C13=C14 bond. As in the case of BR, the 1,184(−)/1,178(+) cm^−1^ bands are insensitive to the H-D exchange (Figure 16a, dotted line), being thus assignable to C10-C11 stretching vibration [33]. Strong positive peaks at 1,277 and 1,204 cm^-1^ in H_2_O and at 1,231 cm^-1^ in D_2_O were also observed for the 13-*cis* form of BR, where positive peaks at 1,205 cm^-1^ in H_2_O and at 1,234 cm^-1^ in D_2_O were assigned to be C14-C15 stretching vibrations [33]. Therefore, corresponding peaks at 1,204 cm^-1^ in H_2_O and at 1,231 cm^-1^ in D_2_O are assignable to the C14-C15 stretching vibration of 13C-ASR_K_. Spectral coincidence between BR and ASR implies similar chromophore structures of their 13-*cis* forms and respective K states. Hydrogen-out-of-plane (HOOP), N-D in-plane bending, and methyl rocking vibrations are observed in the 1,110-890 cm^-1^ region, and the presence of strong HOOP modes represents distortions of the retinal molecule [60]. The AT-ASR_K_ minus AT-ASR spectra exhibit two strong peaks at 968 and 957 cm^-1^ (Figure 16b).

In contrast, many positive bands were observed in the 13C-ASR_K_ minus 13C-ASR spectra, whose frequencies are at 1,002, 991, 981, 971, 965, and 957 cm^-1^ (Figure 16a). This observation suggests that the chromophore of 13C-ASR_K_ is more distorted along the polyene chain than that of AT-ASR_K_.

**Figure 17.**
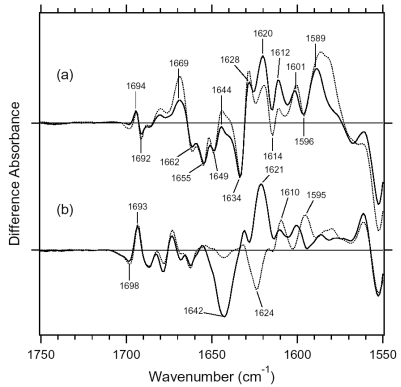
The 13C-ASR_K_ minus 13C-ASR (a) and the AT-ASR_K_ minus AT-ASR (b) spectra (pH 7) in the 1,750–1,550 cm^-1^ region, mostly representing vibrations of the protein moiety. The sample was hydrated with H_2_O (solid lines) or D_2_O (dotted lines). One division of the y-axis corresponds to 0.0025 absorbance units. This figure is reprinted with permission from Kawanabe *et al.* [59]. Copyright 2006 American Chemical Society.

Figure 17 shows the 13C-ASR_K_ minus 13C-ASR (a) and the AT-ASR_K_ minus AT-ASR spectra (b) in the 1,750–1,550 cm^-1^ region. Amide I vibrations appear in this frequency region together with the C=N stretching vibration of the protonated retinal Schiff base. In general, the former is insensitive to the H-D exchange, whereas the latter exhibits downshift in D_2_O. In the case of AT-ASR, a prominent peak pair at 1,642(−) and 1,621(+) cm^-1^ is assignable to the C=N stretchings of AT-ASR and AT-ASR_K_, respectively, because of the spectral shifts to 1,624(−) and 1,610(+) cm^-1^ in D_2_O (Figure 17b). In fact, we observed the downshift of the bands at 1,642(−) and 1,621(+) cm^-1^ by 10 cm^-1^ for [ζ-^15^N] lysine-labeled ASR, indicating that they originate from the C=N stretching vibrations (data not shown). It should be noted that the spectral changes of amide I vibrations at 1,660–1,630 cm^-1^ are small in AT-ASR_K_ minus AT-ASR, which is clearly seen in D_2_O (Figure 17b, dotted line), suggesting that no structural changes of the peptide backbone occur in AT-ASR upon retinal isomerization. The spectral features are quite different for 13C-ASR. Figure 17a shows the presence of the H-D exchange independent bands in the 1,660–1,630 cm^-1^ region, located at 1,669(+), 1,662(−), 1,655(−), 1,649(−), 1,644(+), 1,634(−) and 1,628(+) cm^-1^. This suggests perturbation of the peptide backbone upon retinal photoisomerization of 13C-ASR. In particular, the peaks at 1,662, 1,655, and 1,649 cm^-1^ are ascribable to the amide I vibrations of the α-helix. Helical perturbation may be correlated with many peaks of the HOOP vibrations in 13C-ASR_K_.

Unlike in AT-ASR (Figure 17b), the 13C-ASR_K_ minus 13C-ASR spectra (Figure 17a) do not show H-D exchange dependent bands clearly. This indicates that the C=N stretching vibrations are not clearly observed in the spectra. Reproducible differences between H_2_O and D_2_O samples in Figure 17a suggest that the C=N stretching vibrations are present in this frequency region. In fact, bands at 1,640–1,620 cm^-1^ were sensitive to [ζ-^15^N]lysine labeling (not shown). However, the absence of clear peaks of the C=N stretch requests spectral analysis using double difference spectra. The C=N stretching vibrations have been regarded as an important marker, because the difference in frequency between H_2_O and D_2_O samples probes hydrogen-bonding strength of the Schiff base [39,40]. In the present study, however, we discuss the hydrogen-bonding strength of the Schiff base by use of the N-D stretching in D_2_O (see below), which is the more direct probe [41].

In the carboxylic C=O stretching frequency region (>1,700 cm^-1^), there are no bands for 13C- and AT-ASR_K_ (Figures 16 and 17). This implies that Asp and Glu residues are located far from the retinal even if they are protonated. In the BR_K_ minus BR difference spectra, the bands at 1,742(-) and 1,733(+) cm^-1^ were assigned to the C=O stretching vibrations of the protonated Asp115 [42]. ASR has a glutamine residue (Gln109) at the corresponding position, whose vibrational bands are probably observed at 1,698(-) and 1,693(+) cm^-1^ for AT-ASR (Figure 17b). Similar bands were also observed at 1,704(−) and 1,700(+) cm^-1^ in the difference spectra of *p*pR, which has an asparagine residue at the corresponding position [43]. Therefore, it can be suggested that the structural changes occurring around Asp115 in BR are common for ASR, BR, and *p*pR. Figure 17a shows the bands at 1,694(+) and 1,692(−) cm^-1^ for 13C-ASR, which can be assigned to the C=O stretch of Gln109. It is likely that the C=O stretching vibrations of Asp115 in BR are dependent on the isomeric form as well.

### 3.2. S-H Stretching Vibrations of Cysteine Residues

**Figure 18.**
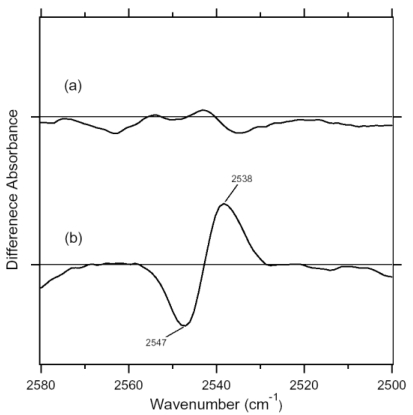
The 13C-ASR_K_ minus 13C-ASR (a) and the AT-ASR_K_ minus AT-ASR (b) spectra (pH 7) in the 2,580–2,500 cm^-1^ region, which correspond to S-H stretching vibrations of cysteine residues. The sample was hydrated with H_2_O. One division of the y-axis corresponds to 0.0003 absorbance units. This figure is reprinted with permission from Kawanabe *et al.* [59]. Copyright 2006 American Chemical Society.

Figure 18 shows the 13C-ASR_K_ minus 13C-ASR (upper panel) and AT-ASR_K_ minus AT-ASR (lower panel) spectra in the 2,580–2,500 cm^-1^ region, which corresponds to S-H stretching vibration of cysteine. As we already reported, there is a negative band at 2,547 cm^-1^ and a positive band at 2,538 cm^-1^ for AT-ASR (Figure 18b). In contrast, no spectral changes were observed for 13C-ASR, indicating that the 13-*cis* to all-*trans* isomerization in ASR does not alter the local structure of cysteines (Figure 18a). We suggested that the observed vibrational bands may be assignable to the S-H stretching of Cys203 in previous section [20].

### 3.3. Assignment of the N-D Stretching Vibrations in 13C-ASR and AT-ASR

**Figure 19.**
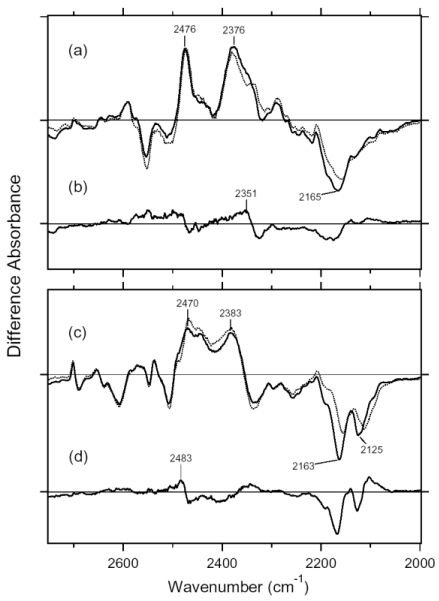
The 13C-ASR_K_ minus 13C-ASR (a) and the AT-ASR_K_ minus AT-ASR (c) spectra (pH 7) in the 2,750–2,000 cm^-1^ region for [ζ-^15^N]lysine-labeled (dotted line) and unlabeled (solid line) ASR. Double difference spectra in (a) and (c) (solid line minus dotted line) are shown in (b) and (d), respectively. The samples were hydrated with D_2_O, and spectra were measured at 77 K. One division of the y-axis corresponds to 0.0007 absorbance units. These figures are reprinted with permission from Kawanabe *et al.* [59]. Copyright 2006 American Chemical Society.

X-D stretching vibrations of protein and water molecules appear in the 2,750–2,000 cm^-1^ region for the films hydrated with D_2_O (Figure 19). The solid line of Figure 19c shows the AT-ASR_K_ minus AT-ASR spectrum reported earlier [20]. On the other hand, the 13C-ASR_K_ minus 13C-ASR spectrum (solid line of Figure 19a) is also obtained previously [59].

Since the N-D stretching vibrations of the Schiff base should be present in this frequency region, we then attempted to assign them by use of the [ζ-^15^N]lysine-labeled ASR sample. Figure 19a compares the 13C-ASR_K_ minus 13C-ASR spectra between [ζ-^15^N]lysine-labeled (dotted line) and unlabeled (solid line) ASR. Clear isotope-induced spectral downshift was observed for intense positive and negative bands at 2,376 and 2,165 cm^-1^, respectively. Other bands are identical between [ζ-^15^N]lysine-labeled and unlabeled 13C-ASR. Thus, we are able to conclude that the N-D stretching vibrations of the Schiff base are present in this frequency region. It should however be noted that the strong positive peak at 2,376 cm^-1^ probably contains other vibrations because the isotope effect was observed in the broad range of 2,370–2,320 cm^-1^ (Figure 19a).

In fact, the AT-ASR_K_ minus AT-ASR spectra contain such peak at 2,383 cm^-1^ as well (Figure 19c), which may originate from amide A vibrations. By use of double difference spectra from the data shown in Figure 19a, we determined that the N-D stretching vibration of the Schiff base in 13C-ASR_K_ is located at 2,351 cm^-1^ (Figure 19b).

Figure 19c compares the AT-ASR_K_ minus AT-ASR spectra between [ζ-^15^N]lysine-labeled (dotted line) and unlabeled (solid line) ASR. Clear isotope-induced spectral downshift was observed for the two negative bands at 2,163 and 2,125 cm^-1^, indicating that the bands originate from N-D stretching vibrations of the Schiff base in AT-ASR. Additionally, the positive spectral feature at 2,470 cm^-1^ exhibits isotope shift from [ζ-^15^N]lysine labeling as well. By use of double difference spectra from the data shown in Figure 19c, we determined that the N-D stretching vibration of the Schiff base in AT-ASR_K_ is located at 2,483 cm^-1^ (Figure 19d). The positive peak at 2,470 cm^-1^ probably contains other vibrations such as the O-D stretching vibrations of Thr79. In BR, the O-D frequencies of Thr89, the homologue of Thr79 in ASR, are 2,507 and 2,466 cm^-1^ for BR and BR_K_, respectively [49]. A similar positive band was also observed at 2,476 cm^-1^ for 13C-ASR (Figure 19a).

Thus, by use of [ζ-^15^N]lysine-labeled ASR, we identified the N-D stretching vibrations of the Schiff base at 2,163 and 2,125 cm^-1^ for AT-ASR and at 2,165 cm^-1^ for 13C-ASR. This indicates that the hydrogen-bonding strength is very similar for the two isomeric forms, being slightly stronger in AT-ASR. The X-ray crystallographic structure reported the presence of a water molecule in contact with the Schiff base, making it a good candidate for the hydrogen-bonding acceptor [60]. Similarity of the hydrogen bonding in AT-ASR and 13C-ASR is consistent with the X-ray structure.

We also identified the N-D stretching vibration of the Schiff base at 2,483 cm^-1^ for AT-ASR_K_ and at 2,351 cm^-1^ for 13C-ASR_K_. Upshifted N-D frequencies indicate that retinal isomerization weakens the hydrogen bond of the Schiff base for both AT-ASR and 13C-ASR. Nevertheless, unlike in the unphotolyzed states, the difference in frequencies for the K states implies the different isomerization outcomes for AT-ASR and 13C-ASR. In case of AT-ASR, the upshift of the frequency is >300 cm^-1^, indicating that the hydrogen bond is significantly weakened (or broken) in AT-ASR_K_, presumably because of the rotational motion of the Schiff base. In contrast, the upshift of the frequency is about 200 cm^-1^ for 13C-ASR. This fact suggests that the rotational motion of the Schiff base that accompanies retinal isomerization is smaller in 13C-ASR than in AT-ASR.

### 3.4. O-D Stretching Vibrations of Water in 13C-ASR and AT-ASR

A spectral comparison between the samples hydrated with D_2_O and D_2_^18^O identifies O-D stretching vibrations of water molecules which change their frequencies upon retinal photoisomerization. We showed the absence of the water O-D stretch at <2,500 cm^-1^ for AT-ASR in previous section [20]. This observation was entirely different from the case of BR, being consistent with the correlation between strongly hydrogen-bonded water molecules and proton pumping activity [27].

**Figure 20.**
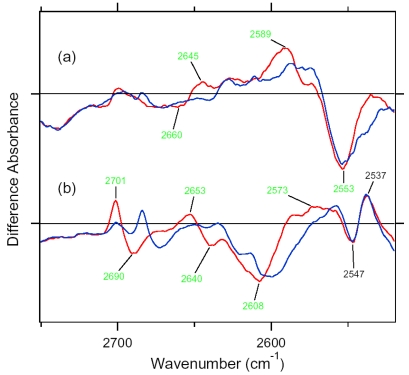
The 13C-ASR_K_ minus 13C-ASR (a) and the AT-ASR_K_ minus AT-ASR (b) spectra (pH 7) in the 2,750–2,520 cm^-1^ region measured at 77 K. Sample was hydrated with D_2_O (red line) or D_2_^18^O (blue line). Green-labeled frequencies correspond to those identified as water stretching vibrations. One division of the y-axis corresponds to 0.0004 absorbance units. This figure is reprinted with permission from Kawanabe *et al.* [59]. Copyright 2006 American Chemical Society.

In this study, we also looked for the water bands in the 13C-ASR_K_ minus 13C-ASR spectrum, but no water bands were found at <2,500 cm^-1^ similar to AT-ASR (data not shown). This fact indicates that the bridged water molecule between the protonated Schiff base and Asp75 forms a weak hydrogen bond for both the all-*trans* and 13-*cis* form. Figure 20 shows difference FTIR spectra in the 2,750–2,520 cm^-1^ region, where weakly hydrogen-bonded water molecules are observed. Green-tagged bands in Figure 20 are assignable to the O-D stretching vibrations of water because of the isotope shift. Figure 20b shows that three negative peaks at 2,690, 2,640, and 2,608 cm^-1^ were assignable to the O-D stretching vibrations of water in AT-ASR, while the bands at 2,701, 2,653, and 2,573 cm^-1^ were assigned as water stretching vibrations of AT-ASR_K_. The bands at 2,547(−)/2,537(+) cm^-1^ are attributed to the H-D unexchangeable S-H stretching vibration of a cysteine residue as shown in Figure 18b. Figure 20a shows that the bands at 2,660(−) and 2,645 (+) cm^-1^ exhibit isotope shift of water. In addition, clear isotope shift was seen for the positive band at 2,589 cm^-1^. The negative band at 2,553 cm^-1^ also contains water O-D stretch, though the small downshift suggests the presence of vibrations other than that of water. Therefore, two positive and two negative peaks can be assigned as O-D stretches of water in 13C-ASR.

### 3.5. Unique Structure of the 13-cis Form of ASR

In this section, we compared the 13C-ASR_K_ minus 13C-ASR and AT-ASR_K_ minus AT-ASR spectra obtained by means of low-temperature FTIR spectroscopy. The HPLC analysis revealed that the dark-adapted ASR is predominantly in the AT-ASR form (97%). Then, the optimal conditions of light adaptation to accumulate 13C-ASR were established, resulting in accumulation of 78% of the 13-*cis* form. This unique property of ASR raises several questions on how ASR relays the signal to its ASRT and the nature of the signaling state of ASR. If there is a structural difference between 13C-ASR and AT-ASR in the ground state, it might result in different binding affinity of the ASRT for 13C-ASR and AT-ASR. But we cannot exclude a general mechanism in which the M state would be the signaling state as in other sensory rhodopsins. Although the light-adapted ASR contains AT-ASR, the appropriate illumination regime allowed us to obtain the 13C-ASR_K_ minus 13C-ASR spectra without any subtraction of the contribution of the all-*trans* form (Figure 15). The spectral comparison of 13C-ASR and AT-ASR upon the retinal isomerization at 77 K led to detection of the structural changes specific for each isomer. In addition, we revealed the hydrogen-bonding strengths of the Schiff base in each state using [ζ-^15^N]lysine-labeled ASR.

### 3.6. Unphotolyzed State of 13C-ASR

We identified the N-D stretching vibration of the Schiff base at 2,165 cm^-1^ for 13C-ASR (Figure 19a). We also identified the N-D stretching vibration of the Schiff base at 2,163 and 2,125 cm^-1^ for AT-ASR. The similar frequencies in 13C-ASR and AT-ASR indicate that the hydrogen-bonding strength of the Schiff base is nearly identical, being slightly stronger in AT-ASR. In the case of BR, the N-D stretching vibrations of the Schiff base were determined to be at 2,171 and 2,124 cm^-1^ [41]. X-ray crystallographic structures of ASR and BR reported the presence of a water molecule in contact with the Schiff base [7,16]. Therefore, similar hydrogen-bonding strength for 13C-ASR, AT-ASR, and BR suggests that the water molecule is a good hydrogen-bonding acceptor for the protonated Schiff base.

Interestingly, two peaks were observed for the N-D stretch of the Schiff base of AT-ASR (Figure 19c) and BR [20], while only one peak was observed for that of 13C-ASR (Figure 19a). Origins of the two peaks in BR, *p*pR, and AT-ASR have not been well understood. Multiple vibrational modes or structural heterogeneity is a possible source of the two N-D stretches. A single peak of the 13-*cis* form in ASR may be useful for understanding of the nature of this mode.

We previously found that water vibrations are entirely different between AT-ASR and BR, though both possess a water molecule between the Schiff base and its counterion (Asp75 for ASR or Asp85 for BR) [7,16]. Namely, the N-O_water_–O_counterion_ (the Schiff base nitrogen, water oxygen, and oxygen of the counterion) angle is 83° and 106° in ASR and BR, respectively. As the consequence, if the water oxygen fully accepts a hydrogen bond of the Schiff base, the O-H group of water points toward the oxygen of Asp85 in BR, but not toward that of Asp75 in ASR. Such a small difference in the angle can possibly determine the hydrogen-bonding strength of water molecules. We did not observe strongly hydrogen-bonded water molecules for 13C-ASR in this study (Figure 20a). This is consistent with the above argument, because the X-ray crystal structure of ASR provides a similar position of the Schiff base, the water, and Asp75 for both isomers at 2.0 Å resolution [16].

We observed water O-D stretches of 13C-ASR at 2,660 and 2,553 cm^-1^ (Figure 20a), which correspond to O-H stretches at 3,592 and 3,481 cm^-1^, respectively, from the spectral analysis of the O-H stretching vibrations in H_2_O (not shown). The O-H stretches of AT-ASR corresponding to the O-D stretches at 2,690, 2,640, and 2,608 cm^-1^ in Figure 20b are found at 3,636, 3,558, and 3,530 cm^-1^, respectively. Since only the water bridging the Schiff base and Asp75 is located close to the chromophore, it is a reasonable postulation that two water bands of ASR originate from O-D (O-H) stretches of this water molecule. In general, a water molecule has two O-H groups, and their frequencies are distributed in the wide 3,700-2,700 cm^-1^ region depending on their coupling and hydrogen-bonding strength [61]. Gaseous water exhibits asymmetric and symmetric stretching modes at 3,755 and 3,657 cm^-1^, respectively, and the stretching frequency is lowered as its hydrogen bonding becomes stronger [22]. It must be noted that the hydrogen bonding strengths of the two O-H groups are probably not equivalent in the restricted protein environment, which breaks the C_2v_-type symmetry. In such C_S_-type symmetry, one O-H is hydrogen bonded and the other O-H is unbonded, and their frequencies are widely split. That is the case for the bridged water of BR, where such decoupling of the two stretching modes occurs [54]. Consequently, one O-D stretch of water is at 2,171 cm^-1^, while another O-D stretch of water is at 2,636 cm^-1^. We suggested that the former points toward Asp85, while the latter points toward Asp212 [54]. Nonsymmetrical bonding of the water molecule in BR is presumably important for the function [23,26].

**Figure 21.**
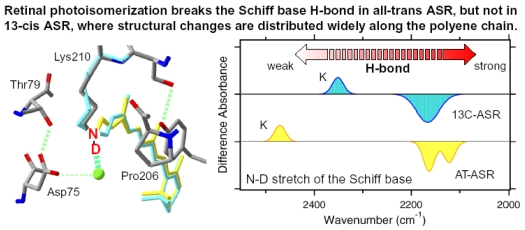
(Left) The X-ray structure around retinal Schiff base. Yellow retinal is all-*trans* form and blue retinal is 13-*cis* form. (Right) The diagram of the ASR_K_ minus ASR infrared spectra in X-D vibration region. It shows only N-D stretch of the Schiff base. This figure is reprinted with permission from TOC of Kawanabe *et al* [59]. Copyright 2006 American Chemical Society.

In the case of 13C-ASR, the frequency difference between the O-D stretches is about 100 cm^-1^. Corresponding O-H stretches are also about 100 cm^-1^ different, being comparable to the gaseous water. Therefore, stretching vibrations of the water molecule are presumably coupled in 13C-ASR, where *anti*-symmetric and symmetric O-D stretches are located at 2,660 and 2,553 cm^-1^, respectively. The situation is probably similar for AT-ASR, where two out of the three bands at 2,690, 2,640, and 2,608 cm^-1^ originate from the O-D stretches of the bridging water. The presence of the additional water band indicates involvement of more distant water upon formation of AT-ASR_K_ (Figure 21).

### 3.7. Photoisomerization Process of 13C-ASR in Comparison with that of AT-ASR

Upon light absorption in 13C-ASR, photoisomerization probably takes place at the C13=C14 (double) bond, leading from the 13-*cis*, 15-*syn* to the all-*trans*, 15-*syn* form. It is generally accepted that the primary K intermediate is a high-energy state for retinal proteins. Chromophore distortion is one of the characteristic features of such high energy state, and HOOP vibrations monitor the chromophore distortion. The appearance of numerous HOOP modes in 13C-ASR_K_ *vs.* just two in AT-ASR_K_ (Figure 16) implies that the chromophore distortion in 13C-ASR_K_ is distributed more widely along the polyene chain. In other words, chromophore distortion is more localized in the Schiff base region for AT-ASR_K_. Such difference in HOOP modes is presumably correlated with the other observations including amide I, cysteine S-H stretch, the Schiff base N-D stretch, and water O-D stretch modes, as discussed below.

Amide I vibrations of the α-helix were clearly observed for the transition from 13C-ASR to 13C-ASR_K_ as shown by the negative bands at 1,662, 1,655, and 1,649 cm^-1^ in Figure 17a. This is reasonable because the retinal chromophore is surrounded by α-helices. In addition, the bands at 1,634(−)/1,628(+) cm^-1^ are also ascribable to amide I vibration. In contrast, fewer structural changes reported by amide I vibrations were observed for the transition from AT-ASR to AT-ASR_K_ as we showed previously [20]. Instead, it was suggested that imide I vibration, possibly due to Pro206, was greatly altered [20]. Several amide I changes observed only for 13C-ASR are consistent with the picture obtained from the HOOP analysis, suggesting that extensive structural changes take place in 13C-ASR_K_.

No structural perturbation was observed for S-H groups of cysteines in 13C-ASR, whereas there is a negative band at 2,547 cm^-1^ and a positive band at 2,538 cm^-1^ for AT-ASR (Figure 18). This indicates that only the all-*trans* to 13-*cis* isomerization leads to the alteration of the local structure of a cysteine in ASR. We previously suggested that among the three cysteines of ASR, Cys203 in helix G is the most likely candidate for this band. Cys203 is near Pro206 and close to the Schiff base region. Replacement of Cys203 by Ala results in a red-shifted λ_max_ (553 nm) relative to the wildtype ASR (unpublished data). This suggests that the Schiff base region is more perturbed in AT-ASR_K_ than in 13C-ASR_K_. The N-D stretching frequency of the Schiff base in 13C-ASR_K_ (2,351 cm^-1^) is lower than that in AT-ASR_K_ (2,483 cm^-1^), though they are similar between 13C-ASR and AT-ASR. We thus assume that the hydrogen bond of the Schiff base is broken in AT-ASR_K_ but not in 13C-ASR_K_. Consequently, the hydrogen-bonding network is destabilized in AT-ASR_K_, and protein structural changes proceed through the network, where the L, M (deprotonation of the Schiff base), and O states can be produced from AT-ASR. In contrast, structural perturbation of the Schiff base region is smaller in 13C-ASR_K_, where the structural changes are distributed more widely.

The number of observed water bands was two for 13C-ASR and three for AT-ASR (Figure 20). As discussed above, the two water bands in 13C-ASR are assignable to the water molecule in the Schiff base region. The presence of an additional water band indicates involvement of more distant water upon formation of AT-ASR_K_. The second nearest water molecule in the X-ray structure is located 8.3 Å from the Schiff base nitrogen in the structure of AT-ASR and 8.0 Å in the structure of 13C-ASR [16]. That water is located between Trp176 and Phe213 in the cytoplasmic region. The third nearest water molecule in the X-ray structure is located 9.2 Å from the Schiff base nitrogen in the structure of AT-ASR and 9.7 Å in the structure of 13C-ASR [16]. That water is located near Arg72 in the extracellular region. No water molecules are present near the polyene chain. Thus, water signals may also be consistent with the above view that the chromophore of 13C-ASR_K_ is distorted more widely along the polyene chain than that of AT-ASR_K_, which has larger changes in the Schiff base region.

In conclusion, ASR accommodates both all-*trans* and 13-*cis*, 15-*syn* retinal in the ground state according to the X-ray crystal structure [16]. On the other hand, the present FTIR study revealed that protein structural changes upon retinal photoisomerization were significantly different between 13C-ASR and AT-ASR. They must trigger global protein structural changes in each photoreaction cycle, resulting in the photochromic behavior. The photochromic signaling mechanism of ASR has not been found, but we should be able to reveal such mechanism if the AT-ASR and 13C-ASR states differ in the binding affinity of the ASRT. The other possibility is that the M state from the photocycle of AT-ASR, which is conformationally changed, would be the signaling state similar to other sensory rhodopsins.

## 4. Photochromism of *Anabaena* Sensory Rhodopsin

Rhodopsins convert light into signal or energy, and retinal is their chromophore molecule [62-64]. The retinal forms a protonated Schiff base linkage (C=NH^+^) with a lysine at the seventh helix in original state.

It is well-known that the protein environment of rhodopsins accommodates the retinal chromophore optimally to its functions. For example, the specific chromophore-protein interaction leads wide color tuning in human visual pigments with a common chromophore (11-*cis* retinal) [65], and protein controls the highly efficient photoisomerization from 11-*cis* to the all-*trans* form in visual rhodopsins [66].

**Figure 22.**
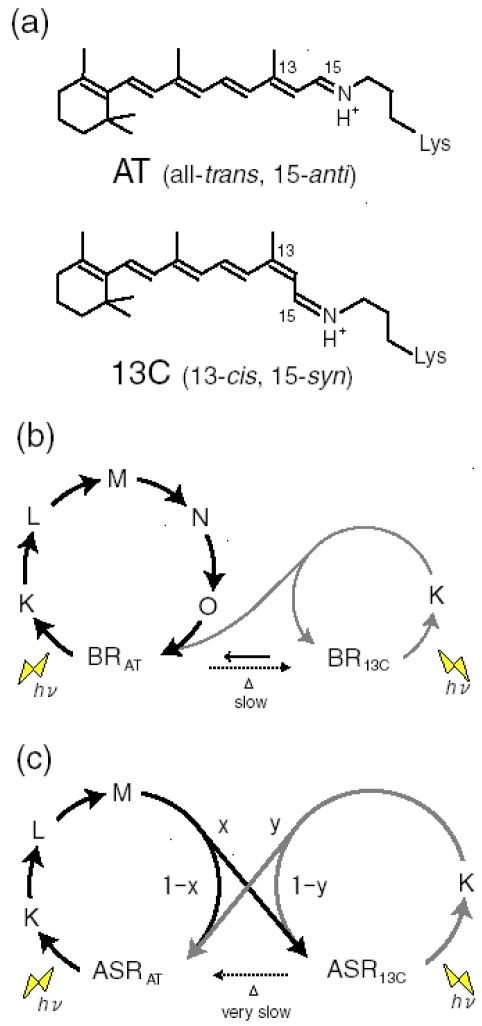
(a). The structure of the retinal chromophore of microbial rhodopsins in the dark. (b). Photo and thermal reaction scheme in a light-driven proton pump bacteriorhodopsin (BR). Only BR_AT_ possesses proton-pump activity, and the reaction of BR_AT_ is 100 % cyclic without any branching reaction into BR_13C_. Dotted arrows represent thermal reaction in the dark. (c). Photo and thermal reaction scheme in *Anabaena* sensory rhodopsin (ASR). While ASR_AT_ is a predominant species in the dark (dotted arrow), photoexcitation of ASR_AT_ and ASR_13C_ yields the reaction of each species, either cyclic or branching, leading to the photocycle or photochromism, respectively. x and y are the branching ratio from ASR_AT_ and ASR_13C_, respectively. These figures are reprinted with permission from Kawanabe *et al* [71]. Copyright 2007 American Chemical Society.

Specific control of retinal photochemistry by protein can be also seen in rhodopsins from halophilic archaebacteria such as the light-driven proton pump bacteriorhodopsin (BR) [5,6,66]. Unlike visual rhodopsins, BR accommodates the retinal chromophore as the all-*trans*,15-*anti* (AT; BR_AT_) and 13-*cis*,15-*syn* (13C; BR_13C_) forms (Figure 22a) [67]. BR_AT_ and BR_13C_ are in equilibrium in the dark, while only BR_AT_ possesses proton-pump activity (Figure 22b). Absorption of light by BR_AT_ yields isomerization to the 13-*cis*, 15-*anti* form, which triggers a cyclic reaction that comprises the series of intermediates, intermediates, K, L, M, N, and O [5,6]. During the photocycle, one proton is translocated from the cytoplasmic to extracellualr side.

Photoexcitation of BR_13C_ partially converts it to BR_AT_, which is called “light-adaptation”, but BR_AT_ is not converted into BR_13C_ photochemically. Photocycle of BR_AT_ with 100% yield is advantageous for repeating the proton-pumping cycle. This is also the case for other proton pumps found in eubacteria (proteorhodopsin) [14] and eucaryotes (*Leptosphaeria* rhodopsin) [68]. In addition, haloarchaeal sensory rhodopsins possess only the AT chromophore in the dark, indicating that its photocycle is important also for light-signal conversion [69,70]. Thus, the photocycle of the AT form with 100% yield has been the common mechanism for the functional processes of microbial rhodopsins.

Recently, a microbial rhodopsin has been discovered in *Anabaena* (Nostoc) PCC7120, which is believed to function as a photoreceptor for chromatic adaptation [15]. In fact, the expected photochromism was found between the AT and 13C forms for *Anabaena* sensory rhodopsin (ASR) [57]. These findings imply strongly branching reactions, from ASR_AT_ to ASR_13C_ and from ASR_13C_ to ASR_AT_ (Figure 22c), in striking contrast to what is known for microbial rhodopsins. Ideally, the conversion ratios should be unity for photochromic reactions (x = y = 1 in Figure 22c), but this is exactly the opposite of the properties of pump rhodopsins, such as BR. X-ray crystal structures reported similar chromophore structures and protein environments for ASR_AT_ [16] and BR_AT_ [7]. Do photochromic reactions indeed take place for ASR_AT_ and ASR_13C_? In this chapter, we determined the branching ratios (x and y values) for ASR_AT_ and ASR_13C_ by means of low-temperature UV-visible spectroscopy. Surprisingly, the obtained x and y values were unity, indicating that the photoreactions of ASR_AT_ and ASR_13C_ are completely photochromic. The complete photochromic reactions are highly advantageous for the chromatic sensor function of ASR.

### 4.1. Photoconversion of ASR_AT_ (1) Photoreaction at 170 K

We first examined the branching ratio of ASR_AT_ (x value in Figure 22c) because previous HPLC analysis revealed that the dark-adapted ASR in PC liposomes contains predominantly (97%) ASR_AT_ [59]. Dark-adapted ASR was illuminated at 170 K, and then warmed to 277 K. The photoconversion yield of ASR_AT_ to its intermediates was calculated using the spectra at 170 K, which was compared with the conversion of ASR_AT_ to ASR_13C_ at 277 K.

**Figure 23.**
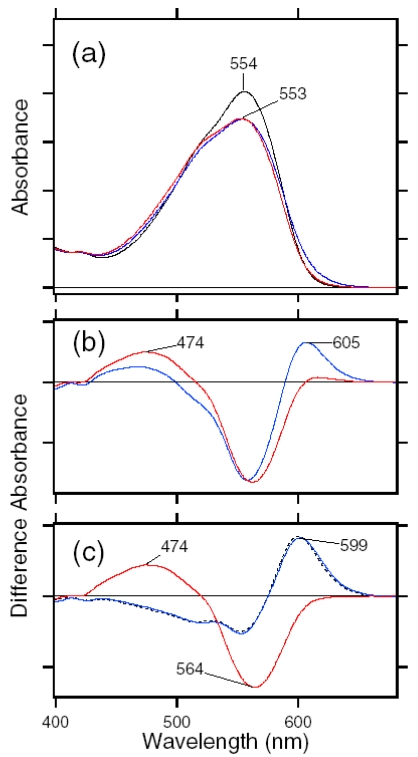
(Left). (a). Absorption spectra of the dark-adapted ASR (black line), illuminated with >580 nm (red line) and 501 nm (blue line) light at 170 K. It should be noted that the dark-adapted ASR corresponds to ASR_AT_, because it contains negligible amount of ASR_13C_ (2.9%) in the present sample conditions [59]. (b). Light-minus-dark difference absorption spectra of ASR with >580 nm (red line) and 501 nm (blue line) light at 170 K. (c). L minus ASR_AT_ (red line) and K minus ASR_AT_ (blue line) difference absorption spectra at 170 K. Black broken line corresponds to the K minus ASR_AT_ spectrum at 130 K, where only the K intermediate is formed. See text for detail. One division of the y-axis corresponds to 0.1(a) and 0.05(b,c) absorbance units. These figures are reprinted with permission from Kawanabe *et al.* [71]. Copyright 2007 American Chemical Society.

The black line in Figure 23a shows the absorption spectrum of the dark-adapted ASR at 170 K (λ_max_ = 554 nm). Illumination at >580 nm (red line) or 501 nm (blue line) resulted in reduction of the peak absorbance and increase of the shorter or longer wavelength tail, indicating the formation of the L and K photointermediates, respectively. Figure 23b shows the corresponding difference spectra, and positive peaks at 474 and 605 nm are characteristic absorption of the L and K intermediates, respectively. On the other hand, no positive band at about 400 nm indicates that the M intermediate is not formed at 170 K.

Since the red and blue spectra in Figure 23b contain contribution of the L and K intermediates, we next obtained the K minus ASR_AT_ and L minus ASR_AT_ spectra. The L minus ASR_AT_ spectrum was obtained by subtracting the blue spectrum from the red one in Figure 23b, so that the spectral shape at about 600 nm coincides with that of the absolute spectrum of the dark-adapted ASR (black line in Figure 23a). The red spectrum in Figure 23c represents the L minus ASR_AT_ spectrum thus obtained. Then, the L minus ASR_AT_ spectrum was subtracted from the blue spectrum in Figure 23b so as to resemble that at 130 K (black dotted line in Figure 23c), where the only photoproduct is the K intermediate. The blue spectrum in Figure 23c represents the resulting K minus ASR_AT_ spectrum. Isosbestic points are at 520 nm between ASR_AT_ and L, and at 575 nm between ASR_AT_ and K.

**Figure 24.**
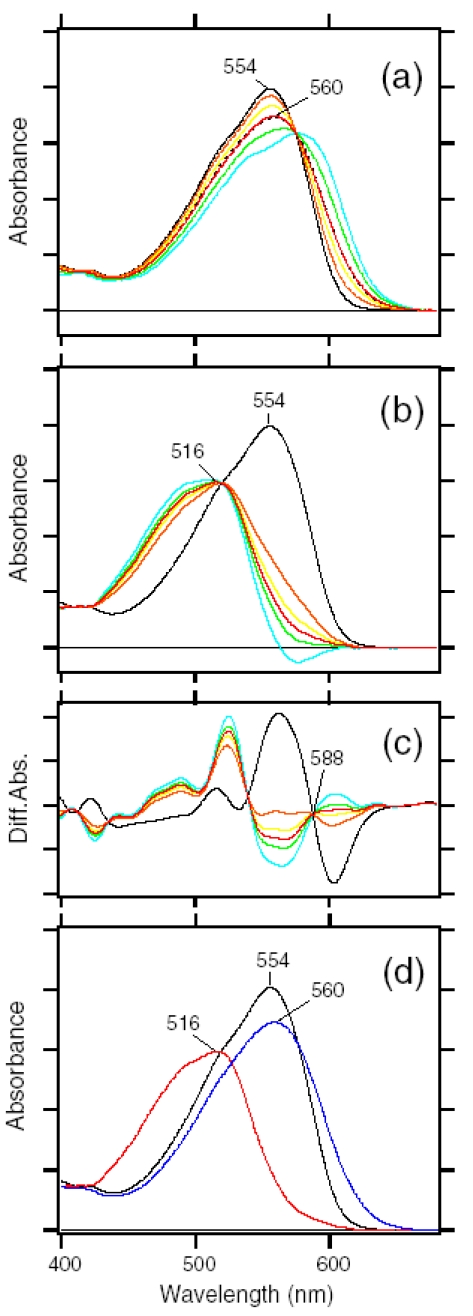
Determination of the absorption spectra of the K and L intermediates of ASR_AT_ at 170 K. (a,b) Black solid line represents absorption spectrum of ASR_AT_ at 170 K. (c) Second derivatives of absorption spectra in Figure 24b, where the corresponding spectra are shown by the same color. (d). Absorption spectra of ASR_AT_ (black line), the K intermediate (blue line) and the L intermediate (red line) at 170 K. The spectra of the K and L intermediates are reproduced from the red spectra in Figure 24a and b, respectively. One division of the y-axis corresponds to 0.1 (a,b,d) and 0.0002 (c) absorbance units. These figures are reprinted with permission from Kawanabe *et al.* [71]. Copyright 2007 American Chemical Society.

We then determined the absorption spectra of the K and L intermediates of ASR_AT_ at 170 K. Absorption spectrum of the K intermediate can be obtained from the K minus ASR_AT_ difference spectrum (blue line in Figure 23c) and photoconversion ratio from ASR_AT_ to the K intermediate. Absorption spectrum of the L intermediate can be obtained from the L minus ASR_AT_ difference spectrum (red line in Figure 23c) and photoconversion ratio from ASR_AT_ to the L intermediate. Five colored lines in Figures 24a or b correspond to the calculated spectra of the K intermediate of ASR_AT_ or the L intermediate of ASR_AT_ at various percentages of conversion (100-17% from orange to blue; 32% for the red line, respectively).

**Figure 25.**
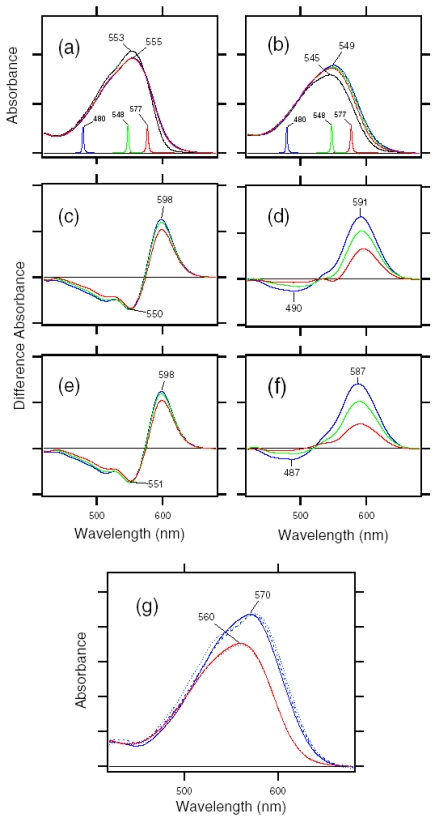
Determination of the absorption spectra of the K intermediates of ASR_AT_ and ASR_13C_ at 130 K. Black solid line represents the absorption spectra of the dark-adapted (a) and light-adapted (b) ASR at 130 K. Blue, green, and red spectra are those by illumination with a 480, 548, and 577 nm light through interference filters (sharp colored peaks in the figure), respectively. (c) and (d). Light-minus-dark difference absorption spectra of the dark-adapted (c) and light-adapted (d) ASR by illumination with 480 (blue), 548 (green), and 577 (red) nm lights at 130 K. (e) and (f). K-minus-ASR_AT_ (e) and K-minus-ASR_13C_ (f) difference absorption spectra by illumination with 480 (blue), 548 (green), and 577 (red) nm lights at 130 K. Blue solid, dotted, and broken lines represent the spectra of the K intermediate of ASR_13C_ obtained from the blue and red, green and red, and blue and green spectra in f. We regarded the red and blue solid lines as the spectra of the K intermediates of ASR_AT_ and ASR_13C_, respectively. One division of the y-axis corresponds to 0.2 (a,b), 0.04 (c,d,e,f) and 0.1 (g) absorbance units. These figures are reprinted with permission from Kawanabe *et al.* [71]. Copyright 2007 American Chemical Society.

The broken black line in Figure 24a represents the absorption spectrum of the K intermediate of ASR_AT_ at 130 K, which was determined by illuminating ASR_AT_ at two wavelengths as described in Figure 25. Since the red spectrum in Figure 24a coincided well with the black broken line, we regard the red one as the absorption spectrum of the K intermediate of ASR_AT_ at 170 K. On the other hand, the absorption spectrum of the L intermediate was determined from the spectral analysis of the second derivatives of the absorption spectra in Figure 24b. The second derivatives in Figure 24c show that the red spectrum coincides with the zero line at >588 nm. We assume that the L intermediate does not contain spectral component in the second derivative at >588 nm. Consequently, we regarded the red one in Figure 24b as the absorption spectrum of the L intermediate of ASR_AT_ at 170 K. Blue and red spectra in Figure 24d correspond to the absolute spectra of the K and L intermediates, respectively.

Figure 25 shows the determination of the absorption spectra of the K intermediates of ASR_AT_ and ASR_13C_ at 130 K. These spectra were calculated for each illumination wavelengths from the spectra in Figure 25c and d by taking account of the isomeric compositions of the dark-adapted (97% ASR_AT_ and 3% ASR_13C_) and light-adapted (22% ASR_AT_ and 78% ASR_13C_) ASR. The almost identical spectra in Figure 25c and e indicate that the dark-adapted state can be regarded as ASR_AT_. In Figure 25g to determine absorption spectrum of an intermediate, the photoconversion ratio from the unphotolyzed state to the intermediate must be obtained. Such a ratio can be obtained by illuminations at two wavelengths if the quantum yields are independent of wavelength [71]. For instance, ASR_AT_ is illuminated at 480 nm or 577 nm under photoequilibrium conditions:

$$\left(1-{\text{x}}_{1})\;\text{Abs}({\text{ASR}}_{AT},480\;\text{nm})={\text{x}}_{1}\;\phi \;\text{Abs}({\text{ASR}}_{\text{AT}}(\text{K}),480\;\text{nm}\right)$$

$$\left(1-{\text{x}}_{2})\;\text{Abs}({\text{ASR}}_{\text{AT}},577\;\text{nm})={\text{x}}_{2}\;\phi \;\text{Abs}({\text{ASR}}_{\text{AT}}(\text{K}),577\;\text{nm}\right)$$

where x_1_ and x_2_ are relative amount of ASR_AT_(K) in the photosteady state mixture, *φ* is the relative quantum yield of ASR_AT_(K) to ASR_AT_. Abs(ASR_AT_, 480 nm) and Abs(ASR_AT_(K), 480 nm) are the absorbance of ASR_AT_ and the K intermediate at 480 nm, respectively. On the other hand, the following equations are derived from the difference spectra before and after illumination:

$$\Delta \text{Abs}(480\;\text{nm})={\text{x}}_{1}\;(\text{Abs}({\text{ASR}}_{\text{AT}}(\text{K}),480\;\text{nm})-\text{Abs}({\text{ASR}}_{\text{AT}},480\;\text{nm}))$$

$$\Delta \text{Abs}(577\;\text{nm})={\text{x}}_{2}\;(\text{Abs}({\text{ASR}}_{\text{AT}}(\text{K}),577\;\text{nm})-\text{Abs}({\text{ASR}}_{\text{AT}},577\;\text{nm}))$$

where ΔAbs(480 nm) and ΔAbs(577 nm) are the difference absorbances at 480 and 577 nm, respectively. From the blue (480 nm) and red (577 nm) spectra in Figure 25e, absorption spectrum of the K intermediate of ASR_AT_ can be determined by obtaining x_1_ and x_2_ values (red solid line in Figure 25g). Red dotted line corresponds to the spectrum of the K intermediate of ASR_AT_ obtained from the green (548 nm) and red (577 nm) spectra in Figure 25e. Red solid and dotted spectra in Figure 25g are almost identical, implying that quantum yields are wavelength independent.

### 4.2. Photoconversion of ASR_AT_ (2) Thermal Relaxation by warming the Sample from 170 K to 277 K

**Figure 26.**
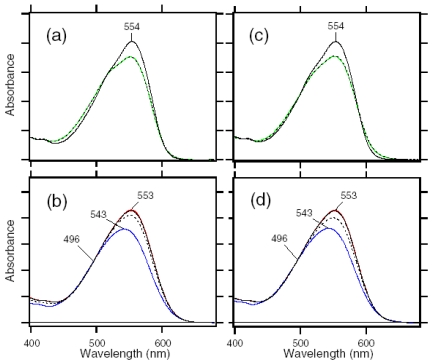
(a) and (c). Absorption spectra of the dark-adapted ASR before (black solid lines) and after (black dotted lines) illuminations with >580 nm (a) or 501 nm (c) light at 170 K. Green lines represent the spectra reconstituted using those in Figure 24d. Under the present illumination conditions, 22 (± 2) and 32 (± 5)% portion were converted into the intermediates in a and c, respectively. (b) and (d). Black solid lines represent absorption spectra of the dark-adapted ASR at 277 K. Red and blue lines correspond to the calculated absorption spectra of ASR_AT_ and ASR_13C_ at 277 K, respectively, which were obtained from those of dark- and light-adapted ASR and the HPLC analysis [59]. Black dotted lines represent absorption spectra at 277 K after illuminations with >580 nm (b) or 501 nm (d) light at 170 K. Under the present illumination conditions, 23 (± 2) and 34 (± 4)% portion were converted from ASR_AT_ to ASR_13C_ at 277 K in b and d, respectively. From a-d, the branching ratio (x in Figure 22c) is obtained to be 1.02 ± 0.13 and 1.10 ± 0.09 for illuminations at >580 nm and 501 nm, respectively, indicating complete branching reactions from ASR_AT_ for both illumination conditions. One division of the y-axis corresponds to 0.1 absorbance units. These figures are reprinted with permission from Kawanabe *et al.* [71]. Copyright 2007 American Chemical Society.

We reconstituted the experimentally obtained spectra (dotted black lines in Figures 26a and c) by use of the spectra in Figure 24d. For the illumination at >580 nm, the dotted black spectrum in Figure 26a coincides well with the sum of 78% ASR, 5% K, and 17% L (green line in Figure 26a), indicating the 22 (±2)% conversion to intermediates at 170 K. On the other hand, for the illumination at 501 nm, the dotted black spectrum in Figure 26c is well coincident with the sum of 68% ASR, 18% K, and 14% L (green line in Figure 26c), indicating the 32 (±5)% conversion at 170 K. We then warmed these states from 170 to 277 K so as to complete the thermal reactions of the K and L states to their end products, and calculated the conversion yield from the spectra. Dotted black lines in Figures 26b and d represent the spectra at 277 K after illumination at >580 and 501 nm, respectively, at 170 K.

Using the absorption spectra of ASR_AT_ and ASR_13C_, the percentages of conversion were calculated to be 23 (±2)% and 34 (±4)% in Figures 26b and d, respectively. The branching ratios (x in Figure 22) were thus determined to be 1.02 ± 0.13 and 1.10 ± 0.09 for illuminations at >580 and 501 nm, respectively. These values demonstrate that the K and L intermediates formed from ASR_AT_ are completely converted into ASR_13C_ without regaining the initial state in a photocyclic reaction.

**Figure 27.**
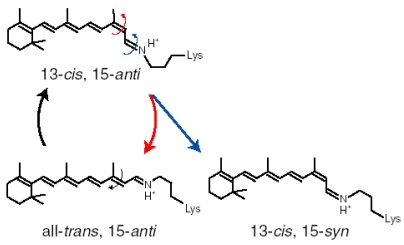
Structural changes of the all-*trans*, 15-*anti* chromophore during photoreactions. The all-*trans*, 15-*anti* form, either in BR_AT_ or ASR_AT_, is first photoconverted to the 13-*cis*, 15-*anti* form, followed by thermal isomerization at C13 = C14 or C15 = N position in BR or ASR, respectively. Such thermal relaxations lead to 100% photocyclic and photochromic reactions for BR_AT_ and ASR_AT_, respectively. This figure is reprinted with permission from Kawanabe *et al.* [71]. Copyright 2007 American Chemical Society.

By means of low-temperature FTIR spectroscopy, we had previously suggested that the primary photoproduct of ASR_AT_ is the 13-*cis*, 15-*anti* form as in BR (Figure 27) [20]. In BR, thermal isomerization takes place at the C13 = C14 bond with virtually 100% yield, recovering the original AT state. In contrast, in ASR thermal isomerization is likely to occur at the C15 = N bond following photoisomerization of ASR_AT_, which converts to the 13C state with 100% yield (Figure 27).

### 4.3. Photoconversion of ASR_13C_ (1) Relative Photoconversion Yields of ASR_AT_ and ASR_13C_ at 277 K

What is the branching ratio (y value in Figure 22c) from ASR_13C_? Unlike ASR_AT_ that is present as nearly the only state in dark-adapted ASR, ASR_13C_ is present in a mixture with ASR_AT_. Therefore, we attempted to determine the branching ratio on the basis of relative photoconversion yields. A previous study showed that the dark-adapted or light-adapted ASRs in PC liposomes possess 97.1% ASR_AT_ and 2.9% ASR_13C_ or 22.1% ASR_AT_ and 77.9% ASR_13C_, respectively [59]. Since ASR_AT_ has greater extinction than ASR_13C_ at 500–600 nm (Figures 26b and d), illumination of the dark-adapted ASR yields an absorption decrease in this wavelength region. In contrast, illumination of light-adapted ASR results in the increase of absorption at 500–600 nm, as reported previously [16]. The isosbestic point of ASR_AT_ and ASR_13C_ is located at 496 nm (Figures 26b and d).

**Figure 28.**
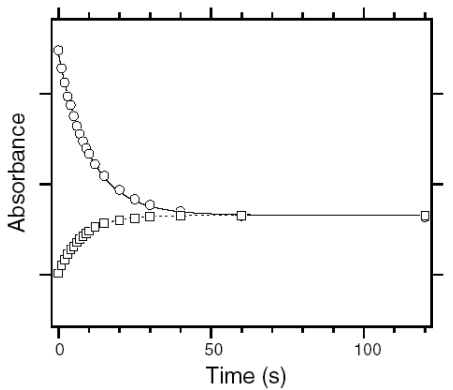
Absorption changes at 569 nm of the dark-adapted (open circles) and light-adapted (open squares) ASR after illumination at the isosbestic point of ASR_AT_ and ASR_13C_ (496 nm) at 277 K. Absorption changes were fitted by single exponentials (solid and broken lines), and the ratio of the initial slope (light-adapted ASR/dark-adapted ASR) was 0.40:1. By taking into accounts of the contents of ASR_AT_ and ASR_13C_ in each state, the ratio between ASR_13C_-to-ASR_AT_ and ASR_AT_-to-ASR_13C_ was determined to be 0.77 (± 0.04). This figure is reprinted with permission from Kawanabe *et al.* [71]. Copyright 2007 American Chemical Society.

In Figure 28, we illuminated the dark-adapted and light-adapted ASR with a 496 nm light at 277 K, and the changes in absorbance at 569 nm (difference absorption maximum between ASR_AT_ and ASR_13C_ at 277 K) were plotted as the function of illumination time.

Thermal conversion from ASR_13C_ to ASR_AT_ is negligible, because it takes 90 min (τ_1/2_) for ASR in PC liposomes at 277 K (data not shown). Absorbance at 569 nm decreases and increases for the dark-adapted and light-adapted ASR, respectively, and both curves eventually coincide after long illumination (Figure 28). The time courses are well fitted by single exponentials, and each photoconversion yield can be obtained from the initial slope (t = 0). By taking into account the contents of ASR_AT_ and ASR_13C_ in the dark-adapted and light-adapted forms, we determined the photoconversion yields ratio between ASR_13C_-to-ASR_AT_ and ASR_AT_-to-ASR_13C_ to be 0.77 (±0.04):1. [Sineshchekov *et al.* estimated a similar photoconversion yield to be 0.3:1 from the HPLC analysis of the photosteady state mixture with white or >520-nm light illumination [57]. While the accurate photoconversion yield is determined by the present method (from the initial slope after illumination at their isosbestic point), such a big difference (0.77 *vs.* 0.3) should be explained. We confirmed that the spectral analysis of the photosteady state, not initial slope, in Figure 28 yields the ratio to be similar (0.75:1). On the other hand, the photoconversion yields ratio between ASR_13C_-to-ASR_AT_ and ASR_AT_-to-ASR_13C_ was significantly reduced by illumination at longer wavelengths, which is close to the value reported by Sineshchekov *et al.* [57]. Thus, the photoconversion yields ratio depends on the illumination wavelength; 0.77 by the 496 nm illumination and about 0.3 by the illumination at >520 nm. We infer that under the photostationary conditions at >520 nm, the intermediate state of ASR_13C_ is photoexcited, presumably forming the original ASR_13C_, while that of ASR_AT_ (the M state) is not. Consequently, ASR_13C_ is accumulated, and the photoconversion yields ratio between ASR_13C_-to-ASR_AT_ and ASR_AT_-to-ASR_13C_ is apparently lowered.] Since the sample is illuminated at the isosbestic point, the ratio is directly correlated with the relative photoconversion yields. Although this value apparently shows a lower branching ratio for ASR_13C_ than for ASR_AT_ (x = 1), it should be noted that the photoisomerization quantum yields are not taken into account in this estimate. A lower photoisomerization quantum yield of ASR_13C_ may provide a lower value for ASR_13C_, and it was indeed the case as shown below.

### 4.4. Photoconversion of ASR_13C_ (2) Relative Photoisomerization Quantum Yields of ASR_AT_ and ASR_13C_ at 130 K

We next compared the relative quantum yields for the photoisomerization of ASR_AT_ and ASR_13C_ by comparing the formation of their K intermediates at 130 K. Since the molar extinction coefficients of their K intermediates are required for the calculation, we determined the absorption spectra of the K-intermediates of ASR_AT_ and ASR_13C_ according to the procedure in Figure 25.

**Figure 29.**
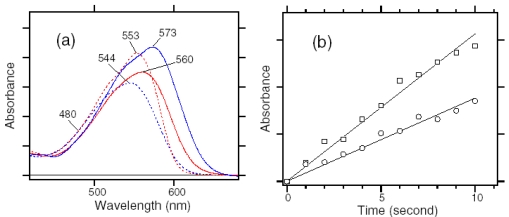
(a). Red and blue broken lines correspond to the absorption spectra of ASR_AT_ and ASR_13C_ at 130 K, respectively, which were obtained from those of the dark- and light-adapted ASR and the HPLC analysis [59]. Isosbestic point is located at 480 nm. Red and blue solid lines represent absorption spectra of the K intermediates of ASR_AT_ and ASR_13C_ at 130 K, which were obtained according to the procedure in Figure 25. (b). Time-dependent absorbance changes of the dark-adapted (open circles) and light-adapted (open squares) ASR. Each sample was illuminated at the isosbestic point at 130 K (480 nm; a), and absorbance changes were monitored at 596 and 590 nm for the dark-adapted and light-adapted ASR, respectively. These figures are reprinted with permission from Kawanabe *et al.* [71]. Copyright 2007 American Chemical Society.

Solid red and blue lines in Figure 29a represent absorption spectra of the K intermediates of ASR_AT_ and ASR_13C_, respectively. Interestingly, the absorption of the K state is decreased for ASR_AT_ but increased for ASR_13C_ increased. Together with the absorption of ASR_AT_ greater than that of ASR_13C_ (broken lines in Figure 29a), this suggests that the 13C *trans* form has a large absorption in the protein pocket of ASR.

**Figure 30.**
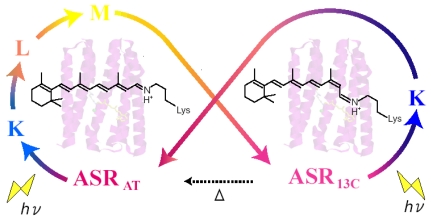
The photoreaction of ASR. Both isomers (ASR_AT_ and ASR_13C_) convert 100% yield to another isomer, respectively. This figure is reprinted with permission from Kawanabe *et al.* [71]. Copyright 2007 American Chemical Society.

We then illuminated the dark-adapted and light-adapted ASR at 480 nm, the isosbestic point of ASR_AT_ and ASR_13C_, at 130 K (Figure 29a). Figure 29b shows time-dependent absorbance changes at each difference absorption maximum (596 and 590 nm) of the dark-adapted and light-adapted ASR. The increase of absorbance is greater for the light-adapted ASR, which contains more ASR_13C_, and originates also from the larger absorbance of the K intermediate of ASR_13C_. By considering the molar extinction coefficients of the K intermediates, the relative quantum yield for the photoisomerization of ASR_13C_ and ASR_AT_ was determined to be 0.73 (±0.07):1. From the data in Figure 26a and c, the branching ratio of ASR_13C_ (y in Figure 22) was therefore determined to be 1.06 ± 0.11. This value demonstrates that the K intermediate formed from ASR_13C_ is completely converted into ASR_AT_ without regaining the initial state in a photocyclic reaction (Figure 30).

### 4.5. Functional Optimization of Photoconversions in Rhodopsins

The present results reveal that the branching reactions take place with 100% efficiency, both from ASR_AT_ and ASR_13C_. Although the present results were obtained for ASR in liposomes, not in native membranes, this characteristic is highly advantageous for a photochromic sensor. On the other hand, the AT form of BR has 100% photocyclic efficiency (Figure 27), which is important for the proton pump. Thus, it is concluded that ASR and BR have been optimized for their functions, presumably during evolution. It is intriguing that the structures of the chromophore and its binding pocket are similar between ASR [16] and BR [7], although their amino acid sequences are not highly homologous (60%). Our FTIR study revealed that hydrogen bond of the Schiff base is similarly strong in ASR and BR, and they are similarly cleaved after retinal photoisomerization [20]. Replacement of aspartate (Asp212 of BR) by proline in ASR (Pro206) is one of the structural differences. Another difference is the hydrogen bonding strength of the water molecule near the Schiff base. BR possesses a strongly hydrogen-bonded water molecule between the Schiff base and its counterion (Asp85), which appears to be a prerequisite for proton-pump function [27]. ASR possesses such a water molecule between the Schiff base and its counterion (Asp75) [16], but its hydrogen bond is much weaker [20]. These small differences may be determinants for distinguishing photocyclic or photochromic reactions. Recently, Sudo and Spudich converted BR into a sensory receptor by mutation of three hydrogen-bonding residues [72]. This finding also suggests that distinct functions are determined by small differences. In addition, the M intermediate is formed during the photoreaction of ASR_AT_, like BR, but Asp75 is not protonated [73], presumably because the proton is conducted toward the cytoplasmic domain [74]. Further structural analysis of photoreaction intermediates will provide a better understanding of the mechanism for thermal relaxation of the photoisomerized chromophore.

## 5. FTIR Study of the L Intermediate of *Anabaena* Sensory Rhodopsin: Structural Changes in the Cytoplasmic Region

The M intermediate with the deprotonated Schiff base is an important state in proton transport and signal transduction. It has been known that the Schiff base proton is transferred to the counterion (Asp85 in BR) if it is deprotonated. In this case, the proton transfer is toward extracellular side. On the other hand, the previous time-resolved FTIR study of ASR by Shi *et al.* reported the proton transfer to Asp217 in the cytoplasmic side [74], though Asp75 works as the counterion of the Schiff base in ASR. No proton transfer to Asp75 was also reported by Bergo *et al.* [73]. This may be reasonable, because another aspartate (Asp212 in BR) is replaced by proline in ASR, and Asp212 plays an important role in the proton transfer in BR [23,75]. On the other hand, Sineshchekov *et al.* reported that the direction of proton transfer was dependent on the sample conditions, where the direction is toward cytoplasmic and extracellular side for C-terminal truncated and full-length ASR, respectively [76]. According to these results, native full-length ASR in *E. coli* cells exhibits proton transfer direction the same as in BR.

**Figure 31.**
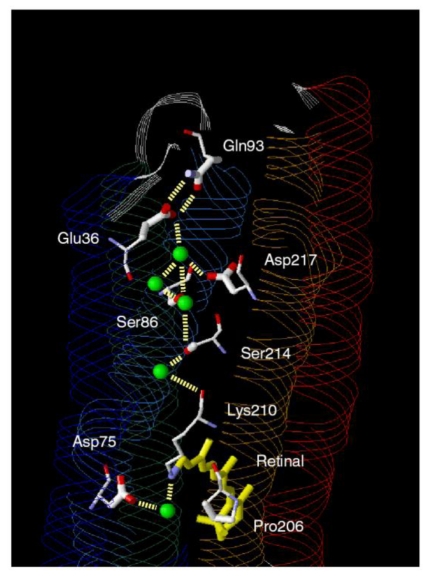
X-ray crystallographic structure of the cytoplasmic region of ASR (PDB entry 1XIO [16]). Top and bottom regions correspond to the cytoplasmic surface and retinal binding pocket, respectively. Green spheres represent water molecules in the cytoplasmic region. Hydrogen-bonds (yellow dashed lines) are inferred from the structure. This figure is reprinted with permission from Kawanabe *et al.* [77]. Copyright 2008 American Chemical Society.

Thus, the molecular mechanism of ASR activation remains yet unclear. In this study, we applied low-temperature FTIR spectroscopy at 170 K to the dark-adapted ASR that has predominantly all-*trans* retinal (97%) [59]. The obtained ASR_L_ minus ASR spectra were similar between the full-length and C-terminally truncated ASR, implying similar protein structural changes for the L state. The ASR_L_ minus ASR spectra were essentially similar to those of BR, but a unique spectral feature was observed in the carboxylic C=O stretching region. The bands at 1,722(+) and 1,703(−) cm^-1^ were observed at pH 5, which was reduced at pH 7 and disappeared at pH 9. The mutation study successfully assigned the bands to the C=O stretch of Glu36. Interestingly, Glu36 is located at the cytoplasmic side, and the distance from the retinal Schiff base is about 20 Å (Figure 31). We also observed pH-dependent frequency change of a water stretching vibration, which is located near Glu36. Unique hydrogen-bonding network in the cytoplasmic domain of ASR will be discussed.

### 5.1. Comparison of the Difference Infrared Spectra of the L Intermediate of Full-length ASR and Truncated ASR in the 1,800–800 cm^-1^ Region

**Figure 32.**
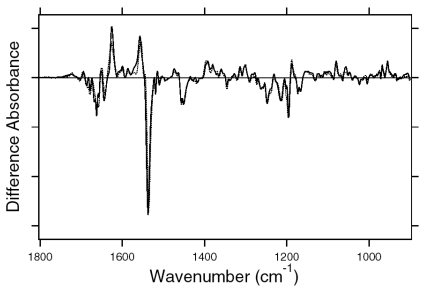
The full-length (solid line) and truncated (dotted line) ASR_L_ minus ASR spectra (pH 7) in the 1,800–900 cm^-1^ region. The spectra are measured at 170 K upon hydration with H_2_O. One division of the y-axis corresponds to 0.004 absorbance units. This figure is reprinted with permission from Kawanabe *et al* [77]. Copyright 2008 American Chemical Society.

The previous photoelectric measurements showed that the direction of charge movement of full-length ASR was different from that of C-terminally truncated ASR (truncated ASR) for the L and M intermediates, whereas both charge movements were similar for the K intermediate [76]. This suggests that full-length and truncated ASR have different structural changes in the L and M intermediates. Therefore, we prepared both full-length and truncated ASR, and measured the difference FTIR spectra for the L intermediate. Figure 32 compares the full-length ASR_L_ minus ASR (solid line) and the truncated ASR_L_ minus ASR (dotted line) spectra at 170 K upon hydration with H_2_O. As is clearly seen, the spectrum of the full-length ASR is very similar to that of the C-terminally truncated ASR. Thus, the present FTIR spectra for the L intermediate showed no effects of the C-terminal truncation. All data below are shown for the full-length ASR including the mutant proteins. It should be noted that we confirmed similarity of the spectra at 170 K between full-length and truncated ASR at acidic and alkaline pH as well, though they could be different at room temperature.

### 5.2. Comparison of the Difference Infrared Spectra of the L Intermediate of ASR and BR in the 1,800–800 cm^-1^ Region

**Figure 33.**
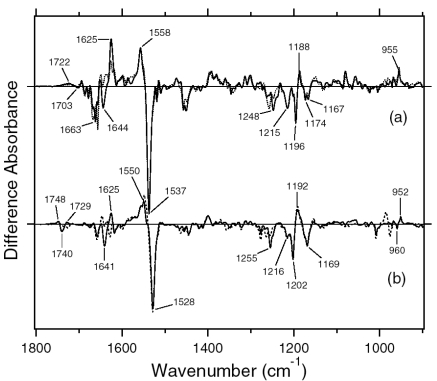
The ASR_L_ minus ASR (a) and the BR_L_ minus BR (b) spectra in the 1,800–900 cm^-1^ region, which are measured at pH 7 and 170 K upon hydration with H_2_O (solid line) and D_2_O (dotted line), respectively. One division of the y-axis corresponds to 0.012 absorbance units. This figure is reprinted with permission from Kawanabe *et al* [77]. Copyright 2008 American Chemical Society.

Figure 33 compares the ASR_L_ minus ASR (a) and the BR_L_ minus BR spectra (b) at 170 K. The samples were hydrated with H_2_O (solid lines) and D_2_O (dotted lines). In Figure 33a, the negative band at 1,537 cm^-1^ corresponds to the ethylenic vibration of all-*trans* retinal in ASR, which exhibits the absorption maximum at 549 nm [59]. The ASR_K_ minus ASR spectrum also showed the negative band at identical frequency [20]. In the case of BR, the ethylenic vibration of the L intermediate is observed at higher frequency (1,550 cm^-1^) than that of the original state (1,528 cm^-1^), which corresponds to the blue-shifted absorption maximum of BR_L_ [29]. Similarly, illumination of ASR results in the spectral upshift to 1,558 cm^-1^. Blue-shifted visible absorption of ASR_L_ is consistent with our low-temperature UV-visible analysis [71].

C-C stretching vibrations of retinal in the 1,300–1,150 cm^-1^ region are sensitive to the local structure of the chromophore. Negative bands at 1,255, 1,216, 1,202, and 1,169 cm^-1^ in Figure 33b were assigned to the C12-C13, C8-C9, C14-C15, and C10-C11 stretching vibrations of BR, respectively [31]. These bands are typical to all-*trans* retinal protonated Schiff base but located at higher frequencies corresponding to charge delocalization in the retinal molecule in BR. BR_L_ has a 13-*cis* retinal, resulting in the appearance of a strong positive band at 1,192 cm^-1^, which is assigned to C10-C11 and C14-C15 stretching vibrations [78]. Essentially similar observation was obtained for ASR. From the similarity in frequency, negative bands at 1,248, 1,215, 1,196, and 1,174 (and/or 1,167) cm^-1^ can be assigned to C12-C13, C8-C9, C14-C15, and C10-C11 stretching vibrations of ASR (Figure 33a).

**Figure 34.**
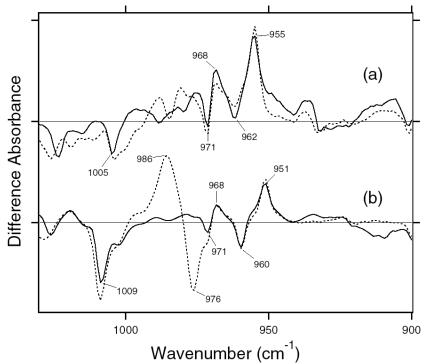
The ASR_L_ minus ASR (a) and the BR_L_ minus BR (b) spectra in the 1,030–900 cm^-1^ region, which correspond to hydrogen-out-of-plane (HOOP) vibrations of the retinal chromophore. The sample was hydrated with H_2_O (solid lines) or D_2_O (dotted lines). One division of the y-axis corresponds to 0.0016 absorbance units. This figure is reprinted with permission from Kawanabe *et al* [77]. Copyright 2008 American Chemical Society.

The difference spectra in the 1,110–890 cm^-1^ region are expanded in Figure 34, where hydrogen-out-of-plane (HOOP), N-D in-plane bending and methyl rocking vibrations appear. The presence of strong HOOP modes represents the distortion of the retinal molecule [60]. The ASR_L_ minus ASR spectra exhibit two positive peaks at 968 and 955 cm^-1^, which possibly correspond to the bands at 968 and 951 cm^-1^ of BR_L_, respectively (Figure 34). The bands at 986(+) and 976(−) cm^-1^ were assigned to the N-D in-plane bending vibrations of BR_L_ and BR, respectively [35]. On the other hand, the ASR_L_ minus ASR spectrum does not show clearer H-D exchange dependent bands in this region than the BR_L_ minus BR spectrum. The 1,009 cm^-1^ band in Figure 34b is insensitive to the H-D exchange and was assigned to the methyl rocking vibration of the retinal in BR. The band at 1,005 cm^-1^ in Figure 34a is also assignable to the methyl rocking vibration in ASR. Thus, similar L spectra were observed for ASR and BR.

Amide-I vibrations appear in the 1,700–1,550 cm^-1^ region together with the C=N stretching vibration of the protonated retinal Schiff base (Figure 33). In general, the former is little sensitive to the H-D exchange, whereas the latter exhibits a downshift in D_2_O. The bands at 1,641(−) and 1,625(+) cm^-1^ were assigned to the C=N stretching vibrations of BR and BR_L_, respectively [35]. In the case of ASR, a prominent negative peak at 1,644 cm^-1^ is assignable to the C=N stretch of ASR, because the D_2_O-sensitive 1,644 cm^-1^ band in the ASR_K_ minus ASR spectra was identified by use of ^15^N-lysine labeled ASR [59]. On the other hand, the C=N stretch of ASR_L_ is not obvious. The positive peak at 1,625 cm^-1^ is a candidate, whereas the downshifted band was not clearly observed in D_2_O (dotted line in Figure 33a). The H-D independent band at 1,663 cm^-1^ presumably originates from amide-I vibration. The frequency suggests structural changes of a distorted α-helix. Since the negative band at 1,663 cm^-1^ is absent for the ASR_K_ minus ASR spectra [20], the structural changes of α-helix newly appear in ASR_L_.

### 5.3. Comparison of the Difference Infrared Spectra of the L Intermediate in Protonated Carboxylic Acid (1,800–1,700 cm^-1^) Region

The infrared difference spectra in this frequency region mainly monitor the structural changes of protonated carboxylic acids. In the BR_L_ minus BR difference spectra, the bands at 1,748(+) and 1,729(+) cm^-1^ were assigned to the C=O stretching vibrations of the protonated Asp96 and Asp115, respectively, while large negative band at 1,740 cm^-1^ contains the corresponding bands of Asp96 and Asp115 in the unphotolyzed state (Figure 33b) [42]. The corresponding amino acids in ASR are Ser86 and Asn105, so that we did not expect any peaks in this frequency region. Nevertheless, Figure 33a shows a broad positive peak at 1,722 cm^-1^ as well as a negative feature at 1,703 cm^-1^, suggesting structural perturbation of carboxylic acids upon formation of ASR_L_. It should be noted that the bands do not originate from the contribution of ASR_M_, because UV-visible spectroscopy confirmed no formation of ASR_M_ at 170 K [71]. The absence of a clear negative band at around 1,400 cm^-1^, characteristic of COO^-^ stretching frequency of negatively charged carboxylates, suggests that appearance of the carboxylic C=O stretch at 1,722 cm^-1^ in ASR_L_ is not due to the newly protonated species, but rather due to the frequency shift from 1,703 cm^-1^ in ASR (Figure 33a).

**Figure 35.**
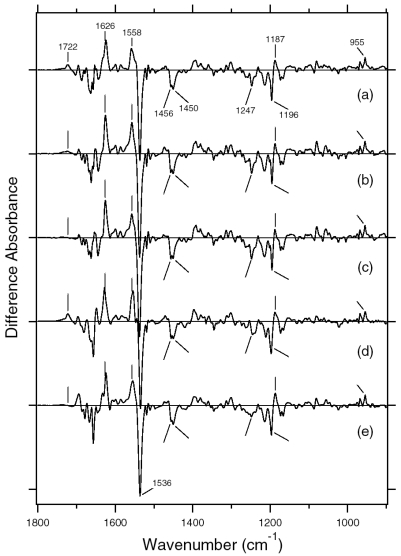
The ASR_L_ minus ASR infrared spectra of the wild type at pH 5 (a), pH 7 (b), and pH 9 (c), D217N at pH 5 (d) and E36Q at pH 5 (e) in the 1,800–900 cm^-1^ region. The spectra are measured at 170 K upon hydration with H_2_O. One division of the y-axis corresponds to 0.009 absorbance units. This figure is reprinted with permission from Kawanabe *et al* [77]. Copyright 2008 American Chemical Society.

**Figure 36.**
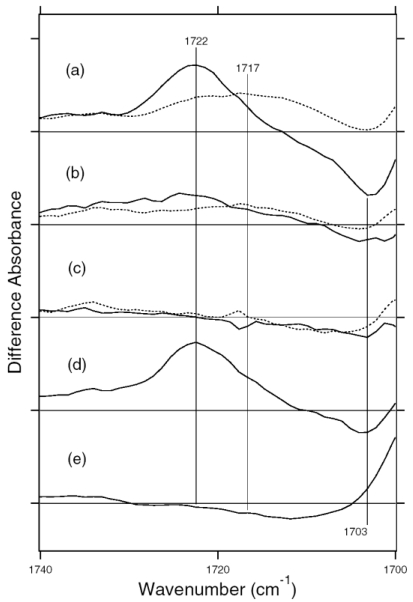
The ASR_L_ minus ASR infrared spectra of the wild type at pH 5 (a), pH 7 (b), and pH 9 (c), D217N at pH 5 (d) and E36Q at pH 5 (e) in the 1,740–1,700 cm^-1^ region. The sample was hydrated with H_2_O (solid lines) or D_2_O (dotted lines). One division of the y-axis corresponds to 0.0008 absorbance units. This figure is reprinted with permission from Kawanabe *et al* [77]. Copyright 2008 American Chemical Society.

To further examine the spectral feature in this region, we measured ASR_L_ minus ASR spectra at acidic (pH 5) and alkaline (pH 9) pH in addition to pH 7 (Figure 33a). We also measured the ASR_L_ minus ASR spectra of D217N and E36Q mutant proteins to identify the responsible carboxylic acid. Figure 35 clearly shows that ASR_L_ is formed at different pH values (5, 7, and 9 in a, b, and c, respectively), as well as for the D217N (d) and E36Q (e) mutants. Figure 36 highlights the carboxylic C=O stretching region, where all spectra were normalized by use of the negative 1,196 cm^-1^ band (Figure 35). Although the positive peak at 1,722 cm^-1^ was broad at pH 7 (Figure 36b), it was enhanced at pH 5 (Figure 36a). In contrast, the 1,722 cm^-1^ band completely disappeared at pH 9 (Figure 36c). Spectral downshift to 1,717 cm^-1^ in D_2_O (Figure 36a) is typical for carboxylic C=O stretching vibrations. Thus, we identify the positive band at 1,722 cm^-1^ in the ASR_L_ minus ASR spectra as a carboxylic C=O stretch, whose pKa was estimated between 6 and 7.

The negative band at 1,703 cm^-1^ exhibits similar pH dependence to that of the 1,722 cm^-1^ band, being enhanced at pH 5, but disappearing at pH 9 (Figure 36a-c). In addition, the 1,703 cm^-1^ band is downshifted in D_2_O (Figure 36a), though the shifted negative band was not clearly observable because of the strong peaks at 1,695(+)/1,687(−) cm^-1^ (Figure 33a). Similar pH dependence strongly suggests that the bands at 1,722(+)/1,703(−) cm^-1^ originate from the same carboxylic group. The absence of pH dependent bands at around 1,400 cm^-1^ (Figure 35) also supports this interpretation.

Finally, the remaining question is the location of the carboxylic group responsible for this spectral feature. The previous time-resolved FTIR study observed a positive carboxylic C=O stretch at 1,716 cm^-1^ in the ASR_M_ minus ASR spectra, and assigned the band to Asp217 located at the cytoplasmic region, because the band disappeared for D217N, but not for E36Q [74]. Interestingly, the 1,716 cm^-1^ band in ASR_M_ was also pH-dependent, whose pKa was between 6 and 7, but the pH dependence was opposite to the present case. Namely, the positive band at 1,716 cm^-1^ was observed at alkaline pH, but not at acidic pH, and the authors interpreted that Asp217 is protonated at acidic pH in the unphotolyzed state [74].

They did not observe frequency change of Asp217 at acidic pH, suggesting no structural changes of Asp217 at acidic conditions. Thus, there has been no information about the C=O stretching frequency of Asp217 at acidic pH, and Asp217 is a possible candidate for the bands at 1,722 (+)/1,703(−) cm^-1^ in the ASR_L_ minus ASR spectra. Here we also measured the spectra of the E36Q mutant, as Glu36 is located near Asp217 (Figure 31). Figures 36d and 36e show the carboxylic C=O stretching region in the ASR_L_ minus ASR spectra of D217N and E36Q, respectively. Similar difference spectra in other frequency regions ensure the normal formation of ASR_L_ for these mutants (Figure 35).

The bands at 1,722(+)/1,703(−) cm^-1^ were reproduced in Figure 36d, indicating that they do not originate from Asp217. On the other hand, the bands at 1,722(+)/1,703(−) cm^-1^ completely disappeared for E36Q (Figure 36e). Thus, we assigned the bands to Glu36. It is generally accepted that the C=O stretching vibrations appear at lower frequency for Glu than for Asp, but the frequency of Glu36 at 1,703 cm^-1^ in ASR is particularly unusual. The frequency is very low, indicating that Glu36 forms a strong hydrogen bond in the unphotolyzed state, which is weakened by structural changes upon formation of ASR_L_ as shown by the upshift to 1,722 cm^-1^.

### 5.4. Comparison of the Difference Infrared Spectra of the L Intermediate in Water O-D Stretching Frequency (2,750–2,500 cm^-1^) Region

**Figure 37.**
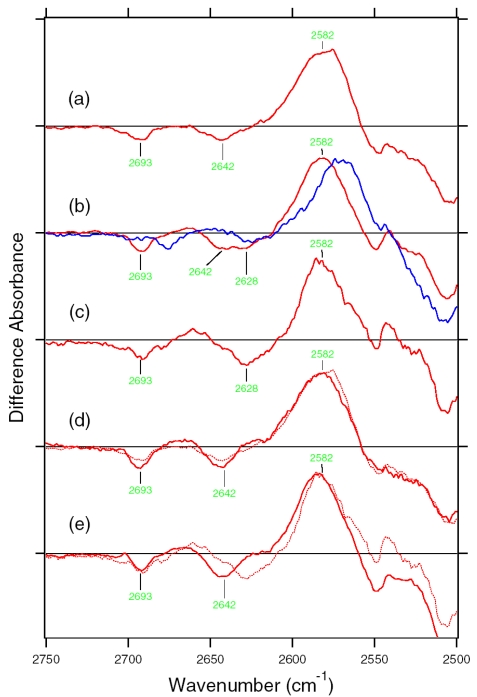
The ASR_L_ minus ASR infrared spectra of the wild type at pH 5 (a), pH 7 (b), and pH 9 (c), E36Q at pH 5 (d) and pH 9 (e) in the 2,750−2,500 cm^-1^ region on red spectra. The spectra are measured at 170 K upon hydration with D_2_O. Blue spectrum is hydrated with D_2_^18^O at pH7 and red dotted lines are corresponding to WT spectra at each pH. One division of the y-axis corresponds to 0.0009 absorbance units. This figure is reprinted with permission from Kawanabe *et al.* [77]. Copyright 2008 American Chemical Society.

The ASR_L_ minus ASR spectra clearly show hydrogen-bonding alteration of Glu36. Since there is a water cluster in the cytoplasmic region near Glu36 (Figure 31), detecting water signals in the ASR_L_ minus ASR spectrum is important. It was not easy, because ASR_L_ decays to the 13-*cis* form, not to the original all-*trans* form, and the sample was dark-adapted again before the next measurement. Nevertheless, we successfully measured the spectra in the frequency region of water O-D stretching vibrations in D_2_O. Figures 37a, 37b, and 37c show the ASR_L_ minus ASR spectra of the wild type at pH 5, 7, and 9, respectively.

The spectra show a negative peak at 2,693 cm^-1^ and a positive peak at 2,582 cm^-1^ at all pH. Since the peaks are downshifted upon hydration with D_2_^18^O, they originate from O-D stretching vibrations of water. The negative peaks at 2,642 and 2,628 cm^-1^ in Figure 37b similarly show the isotope shift of water, indicating that they originate from water O-D stretches. Interestingly, a single peak only exists at 2,642 and 2,628 cm^-1^ for the spectra at pH 5 (Figure 37a) and pH 9 (Figure 37c), respectively. This observation implies that the frequency of the water O-D stretch is pH-dependent, being downshifted at higher pH. The pKa is located at about 7, which is coincident with that of Glu36 (Figure 36). Similar pKa of the water O-D stretch at 2,650–2,620 cm^-1^ to that of Glu36 suggests that the water molecule is located near Glu36. This is indeed the case, because the pH dependence of the water O-D stretch is abolished in E36Q. Figure 37d shows identical spectra between the wild type (dotted line) and E36Q (solid line) at pH 5. However, the frequency shift of the negative band from 2,642 cm^-1^ to 2,628 cm^-1^ in the wild type at pH 9 was absent in E36Q (solid line in Figure 37e). This observation suggests that deprotonation of Glu36 at pH > 7 is correlated with the frequency shift of the water O-D stretch from 2,642 cm^-1^ to 2,628 cm^-1^. The straightforward interpretation is that the water directly interacts with Glu36. It should be noted that the water O-D stretch at 2,650-2,620 cm^-1^ represents a weak hydrogen bond, and we presumably monitor the free O-D stretch of a water molecule interacting with Glu36.

### 5.5. The Structure of ASR_L_

In this study, we report the ASR_L_ minus ASR spectra measured by low-temperature FTIR spectroscopy. Although photoinduced current measurements of ASR_L_ and ASR_M_ reported the different direction of charge signal between C-terminally truncated and full-length ASR [76], the present FTIR study showed almost identical ASR_L_ and ASR spectra for them (Figure 32). While the spectral features were essentially similar to those for BR, a unique feature was obtained for the carboxylic C=O stretching frequency region for ASR. The pH-dependent bands were observed at 1,722(+)/1,703(−) cm^-1^ in the ASR_L_ minus ASR spectra, which were assigned to Glu36. pH-dependent water O-D stretching vibrations in D_2_O were also observed at 2,642 and 2,628 cm^-1^ for the unphotolyzed state of ASR at pH 5 and pH 9, respectively. These bands of pKa were estimated to be between 6 and 7. According to the X-ray structure of ASR, Glu36 is located near the cytoplasmic surface (Figure 31), and the distances from the Schiff base nitrogen of the retinal chromophore to the side-chain oxygens of Glu36 are 19.3 and 20.2 Å [16]. The present study clearly shows that formation of ASR_L_ accompanies hydrogen-bonding alteration of Glu36. Since ASR_L_ is formed at 170 K, this fact demonstrates structural alteration propagating over 20 Å at such low temperatures.

Spectral feature of the water signal in the ASR_L_ minus ASR spectrum resembles that in the BR_L_ minus BR spectrum at 2,700–2,500 cm^-1^ [26]. In particular, an intense positive broadband at 2,630–2,550 cm^-1^ (O-H stretch at 3,550–3,450 cm^-1^) has been regarded as a characteristic for the L state of BR [26]. However, our recent time-resolved FTIR spectroscopy clearly showed the absence of the band for BR_L_ at room temperature, and we concluded that such water signal is a low-temperature artifact, or a feature peculiar at low temperature (170 K) where L is stable [79]. This may be also true for ASR. However, it should be noted that the cryotrapped L state is considerably relaxed to the original state in BR [80,81], but decays to the subsequent intermediates in ASR by warming [71]. This suggests different protein dynamics between ASR and BR, but room-temperature FTIR study of ASR is required for further understanding.

### 5.6. Hydrogen-Bonding Structures in the Cytoplasmic Domain of ASR and ASR_L_

**Figure 38.**
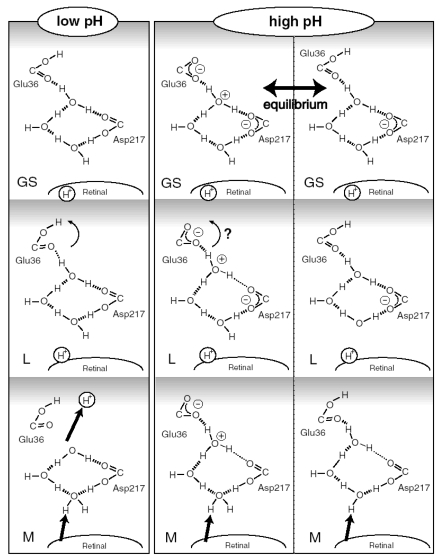
Possible models of hydrogen-bonding alterations in the cytoplasmic region of ASR (see text for details).

The present study showed that the pKa of Glu36 is between 6 and 7 in ASR, and its hydrogen bonding is significantly altered upon formation of ASR_L_. Previous FTIR study reported the pKa of Asp217 being also between 6 and 7 in ASR [74]. According to these observations, Glu36 and Asp217 are both protonated at low pH, while being both deprotonated at high pH. However, the latter may be unlikely, because Glu36 and Asp217 are located close to each other (Figure 31). Here we propose a possible model for activation of ASR based on these observations and X-ray structure [16], shown in Figure 38. It should be noted that the three water molecules constitute a cluster structure in the region of Glu36 and Asp217 (Figure 31), which has to be taken into the account.

At low pH, Glu36 and Asp217 are both protonated in the unphotolyzed state. Hydrogen bond of Glu36 is remarkably strong (C=O stretch at 1,703 cm^-1^), where internal water molecules must play important roles (Figure 38, left panel). Upon ASR_L_ formation, the hydrogen bond of Glu36 is weakened (C=O stretch at 1,722 cm^-1^). The absence of the bands for Asp217 in the ASR_L_ minus ASR spectra (Figure 36) implies that no hydrogen-bonding alteration of this residue occurs. Therefore, we propose that the interaction between Glu36 and the water cluster is weakened, while that between Asp217 and the water cluster is unchanged (Figure 38, left panel). Then, the L-M transition accompanies proton release to the cytoplasmic aqueous phase through the Glu36-Asp217 region.

At high pH, we first postulate that Asp217 is deprotonated according to the previous results [74]. Then, there are two possibilities on the protonation state of Glu36, either deprotonated (Figure 38, middle panel) or protonated (Figure 38, right panel). Figure 38, middle panel shows both Glu36 and Asp217 deprotonated, and the positively charged water cluster (H_3_O^+^ or H_7_O_3_^+^) stabilizes the two negative charges. The structure may resemble the proton release group of BR, where water cluster stabilizes Glu194 and Glu204. Experimental evidence of protonated water cluster was first reported by the group of Dr. Gerwert as a continuum band at 2,200-1,800 cm^-1^ in the room-temperature BR_M_ minus BR spectra [82,83], and we recently identified that the continuum band contains water signal [80]. ASR_L_ formation does not change the hydrogen-bonding network at the cytoplasmic side, because there is no pH-dependent signal at around 1,400 cm^-1^ (Figure 35), but hydrogen-bonding interaction may be altered between Glu36 and the water molecules. According to this model, the L-M transition accompanies proton transfer from the Schiff base to Asp217.

Another model at high pH is based on Glu36 being protonated (Figure 38, right panel). In this case, the pH-dependence can be interpreted not by the direct titration of Glu36, but of other parts of the protein. In addition, hydrogen bond of protonated Glu36 is not changed between ASR and ASR_L_, so that there are no bands in the 1,740–1,700 cm^-1^ region (Figure 36c). ASR_L_ formation does not change the hydrogen bond of Asp217, because there is no pH-dependent signal at around 1,400 cm^-1^ (Figure 35). The L-M transition accompanies proton transfer from the Schiff base to Asp217. At this moment, we cannot exclude either of the two models at high pH. The latter (Figure 38, right panel) may be less plausible, because it predicts no hydrogen-bonding alterations in the cytoplasmic region between ASR and ASR_L_. On the other hand, the two model structures may be in equilibrium. In this regard, detecting water signals in the ASR_L_ minus ASR and ASR_M_ minus ASR spectra is very important, though it is not easy because of the photochromic nature of ASR [71]. The spectral analysis of water is our future focus.

### 5.7. Characteristic Features of Photoreaction in ASR

By use of low-temperature UV-visible spectroscopy, we recently revealed that the stable photoproduct of the all-*trans* form is 100% 13-*cis*, and that of the 13-*cis* form is 100% all-*trans* [71]. This was entirely unique for archaeal-type rhodopsins, because functionally important states known so far were only derived from the all-*trans* form, and the photocycle of the all-*trans* form without branching into the 13-*cis* stable states has been the common mechanism. The complete photocycling for the proton pump in BR and the complete photochromism for the chromatic sensor of ASR are highly advantageous for their functions. Although the protein structures are similar between ASR and BR [16], the present study suggests that the migration of protons to the cytoplasmic side is correlated with the unique photoreactions of ASR.

ASR has Asp75 as a counterion of the retinal chromophore, which corresponds to Asp85 in BR. Nevertheless, the Schiff base proton is transferred not to Asp75 [73,74], but to Asp217 in the cytoplasmic region. What is the mechanism of proton transfer in the opposite direction? The present FTIR spectroscopy of the L intermediate revealed similar structural changes for the chromophores of ASR and BR, suggesting the importance of the surrounding protein moiety. It should be noted that Asp212 in BR is replaced by proline (Pro206) in ASR. Previous studies reported the important role of Asp212 during the M formation, and we proposed a hydration switch mechanism as the primary cause of proton transfer reaction in BR. In this mechanism, the bridged water molecule between the Schiff base and Asp85 forms a strong hydrogen bond transiently, which leads to the proton transfer to Asp85 [20]. Lack of aspartate at position 206 would be significant for ASR. In this regard, we found the absence of strongly hydrogen-bonded water molecules in ASR [20]. Since there is a positive correlation between the strongly hydrogen-bonded water molecules and the proton pumping activity, weakly hydrogen-bonded water molecules in the Schiff base region may be the key element. Interestingly, the replacement of Pro206 to Asp was not sufficient for ASR to function as a BR-like proton pump [84]. Since the Schiff base proton is transferred to the cytoplasmic side, ASR is a very good model system to study the general mechanism of proton pumps in archaeal-type rhodopsins.

## 6. Experimental Section

### ASR Sample Preparation

In the present study, we prepared *C*-terminally truncated and full-length ASR according to the method described previously [15,71,84]. The E36Q and D217N mutants were designed based on the full-length ASR, which were produced by a two-step megaprimer PCR method [85], with two oligonucleotides (COSMO, Seoul, Korea): E36Q F-50-CAG TAC CAA TAC CTT GTG GCG ATG- 30 and D217N R-50-GTA AAT TCA GAA AAA CTA AAT C-30. The final PCR products were cloned into plasmid pKJ606 [86], derived from pMS107, by replacing the original insert with *XbaI/NotI* digestion. After ligation the plasmids were transformed in *E. coli* strain DH5α. All of the mutations were confirmed by DNA sequencing (COSMO, Seoul, Korea). *E. coli* strain BL21 (Stratagene) was transformed by introducing pMS107-derivative plasmid [15], which encodes the wild-type, E36Q and D217N opsin, and was grown in 2xYT medium in the presence of ampicillin (50 μg/ml) at 38 °C. Three hours after IPTG induction with addition of 10 μM all-*trans* retinal, pink-colored cells were harvested, sonicated, solubilized by 1% DM, and purified by a Ni^2+^-NTA column. The purified ASR was then reconstituted into PC liposomes by removing the detergent with Bio-Beads, where the molar ratio of the added PC to ASR was 30:1. The liposomes were washed three times with a buffer [2 mM sodium phosphate (pH 7.0)]. A 40 μL aliquot was deposited on a BaF_2_ window of 18 mm diameter and dried in a glass vessel that was evacuated by an aspirator. [ζ-^15^N]Lysine labeled ASR was prepared as was done for [ζ-^15^N]lysine labeled *pharaonis* phoborhodopsin [87].

### 6.1. FTIR Spectroscopy

FTIR spectroscopy was performed as described previously [36]. Since the all-*trans* form is most abundant for the dark-adapted ASR [15], ASR films were kept in the dark for 3 days. Completely dark-adapted ASR was hydrated with H_2_O, D_2_O, or D_2_^18^O before measurements. Then, the sample was placed in a cryostat (DN-1,704, Oxford) mounted with the cell for the FTIR spectrometer (FTS-40, Bio-Rad). The cryostat was equipped with a temperature controller (ITC-4, Oxford), and the temperature was regulated with 0.1 K precision. All the experimental procedures were performed in the dark or under dim red light (>670 nm) before the spectroscopic measurement.

### 6.2. Accumulation of AT-ASR_K_

Photoreactions of the all-*trans* and 13-*cis* forms strongly depend on the illumination wavelength in ASR. It is required that we reduce the extent of the photoreaction of the 13-*cis* form as much as possible. By using a marker band in the fingerprint (1,200–1,100 cm^-1^) region, we established the following illumination conditions at 77 K, where difference spectra depicted the photoreaction of the 13-*cis* form at <20%. Illumination with 543 nm light at 77 K for 1 min converted ASR to ASR_K_. ASR_K_ was reconverted to ASR upon illumination with >590 nm light for 1 min, as evidenced by a mirror image of the difference spectra. Each difference spectrum was calculated from two spectra constructed from 128 interferograms taken before and after the illumination. Twenty-four (H_2_O and D_2_O) or forty-eight (D_2_^18^O) difference spectra were obtained and averaged to produce the ASR_K_ minus ASR spectrum. ASR molecules are randomly oriented in the liposome film, which is confirmed by linear dichroism experiments, so we did not apply dichroic measurements using an IR polarizer. The obtained difference spectra were compared with those for BR with the window tilting angle of 53.5° in the polarized measurement, where all vibrational bands are observed in the highly oriented BR molecule.

### 6.3. Accumulation of 13C-ASR_K_

We established the following conditions for the light adaptation. Hydrated films were illuminated with >560 nm light (O-58 cutoff filter; Toshiba) from a 1 kW halogen-tungsten lamp for 1 min at 4 °C. HPLC analysis showed that the light-adapted ASR possesses 78% 13-*cis* and 22% all-*trans* forms. The sample was cooled for 3 min after the light adaptation to allow for the complete decay of photoproducts.

Light-adapted ASR contains both all-*trans* and 13-*cis* forms, thus, its photoreactions strongly depend on the illumination wavelength. We established the following illumination conditions to obtain the K intermediate of 13C-ASR (13C-ASR_K_) minus 13C-ASR spectra at 77 K. Illumination with 501 nm light for 1 min first converted 13C-ASR to 13C-ASR_K_ together with the conversion of AT-ASR to the K intermediate of AT-ASR (AT-ASR_K_). Nevertheless, subsequent illumination at >560 nm (O-58 cutoff filter; Toshiba) reverted only 13C-ASR_K_ to 13C-ASR, whereas no photoreversion was found for AT-ASR_K_. In Section 2 for AT-ASR, we illuminated AT-ASR_K_ at >590 nm for the photoreversion to AT-ASR. The present result indicates that illumination at >560 nm does not change the photoequilibrium between AT-ASR and AT-ASR_K_. Subsequent illuminations by 501 nm light and >560 nm light do not induce the spectral features of AT-ASR_K_ minus AT-ASR. Each difference spectrum was calculated from the two spectra constructed from 128 interferograms taken before and after the illumination.

### 6.4. Accumulation of ASR_L_

Illumination with >580 nm light at 170 K for 16 min converted the all-*trans* ASR to ASR_L_. Each difference spectrum was calculated from two spectra constructed from 128 interferograms taken before and after the illumination. Three difference spectra obtained in this way were averaged to produce the ASR_L_ minus ASR spectrum. The BR_L_ minus BR spectra were taken from Kandori *et al.* [36].

### 6.5. UV-Visible Spectroscopy

The UV-visible spectra were measured by a UV-visible spectrometer (V-550, JASCO) equipped with a cryostat (OptistatDN, Oxford). The cryostat was equipped with a temperature controller (ITC-4, Oxford), and the temperature was regulated with 0.1 K precision. A previous HPLC study showed that the completely dark-adapted ASR in PC liposomes is in the all-*trans* form predominantly (97.1 ± 0.1%) [59]. On the other hand, illumination of ASR with >560 nm light (O-58 cutoff filter, Toshiba) from a 1-kW halogen-tungsten lamp for 1 min at 277 K yields formation of 77.9(±1.7)% 13-*cis* form [59]. Three to five independent measurements were averaged.

### 6.6. HPLC Analysis

HPLC analysis was performed as described previously [88]. A high-performance liquid chromatograph was equipped with a silica column (6.0 × 150 mm; YMC-Pack SIL). The solvent was composed of 12% (v/v) ethyl acetate and 0.12% (v/v) ethanol in hexane, and the flow rate was 1.0 mL/min. Extraction of retinal oxime from the sample was carried out by hexane after denaturation by methanol and 500 mM hydroxylamine at 4 °C [69]. The molar composition of retinal isomers was calculated from the areas of the peaks in the HPLC patterns. Assignment of the peaks was performed by comparing them with the HPLC pattern from retinal oximes of authentic all-*trans* and 13-*cis* retinals. Three independent measurements were averaged.

## 7. Conclusions and Perspectives

We studied the detail of photoreaction behavior of *Anabaena* sensory rhodopsin (ASR) by means of spectroscopic techniques. The results in each chapter are summarized as follows.

In Section 2, we applied low-temperature FTIR spectroscopy to the all-*trans* form of ASR, and compared the difference spectra at 77 K with those of BR. The K intermediate minus ASR difference spectra show that the retinal isomerizes from the all-*trans* to the distorted 13-*cis* form like BR. The N-D stretching of the Schiff base was observed at 2,163(−) and 2,125(−) cm^-1^, while the O-D stretchings of water molecules were observed in the >2,500 cm^-1^ region. These results indicate that the protonated Schiff base forms a strong hydrogen bond with a water molecule, which is connected to Asp75 with a weak hydrogen bond. This result with ASR supports the working hypothesis by the Kandori group about the strong correlation between the proton pump activity and the existence of strongly hydrogen bonded water molecules in archaeal rhodopsins. Also we discuss the structural reason why the bridged water molecule does not form a strong hydrogen bond in ASR.

We extended the low-temperature spectroscopic study at 77 K to the 13-*cis*, 15-*syn* form of ASR (13C-ASR) (Section 3). HPLC analysis revealed that light-adapted ASR with light >560 nm at 4 °C possesses 78% 13C-ASR, while dark-adapted ASR has AT-ASR predominantly (97%). Then, we established the illumination conditions to measure the difference spectra between 13C-ASR and its K state without subtracting the difference between AT-ASR and its K state. Spectral comparison between 13C-ASR and AT-ASR provided useful information on structure and structural changes upon retinal photoisomerization in ASR. In particular, previous X-ray crystallographic study of ASR reported the same protein structure for 13C-ASR and AT-ASR, whereas the present FTIR study revealed that protein structural changes upon retinal photoisomerization were significantly different between 13C-ASR and AT-ASR. The differences were seen for HOOP modes of the retinal chromophore, amide I, cysteine S-H stretch, the Schiff base N-D stretch, and water O-D stretch modes. These must trigger different global protein structural changes in each photoreaction cycle leading to the observed photochromic behavior.

ASR has been believed to function as a photoreceptor for chromatic adaptation. In this case, branching reactions, from ASR_AT_ to ASR_13C_ and from ASR_13C_ to ASR_AT_ (Figure 22c), are favorable for ASR, but they are in striking contrast to what is known for microbial rhodopsins. Ideally, the conversion ratios should be unity for photochromic reactions (x = y = 1 in Figure 22c), but this is exactly the opposite of the properties of pump rhodopsins, such as BR. X-ray crystal structures reported similar chromophore structures and protein environments for ASR_AT_ [16] and BR_AT_ [7]. Do photochromic reactions indeed take place for ASR_AT_ and ASR_13C_? In Section 4, we determined the branching ratios (x and y values) for ASR_AT_ and ASR_13C_ by means of low-temperature UV-visible spectroscopy. Surprisingly, the obtained x and y values were unity, indicating that the photoreactions of ASR_AT_ and ASR_13C_ are completely photochromic. The complete photochromic reactions are highly advantageous for the chromatic sensor function of ASR.

In Section 5, we applied low-temperature FTIR spectroscopy at 170 K to the dark-adapted ASR that has predominantly all-*trans* retinal (97%). The obtained ASR_L_ minus ASR spectra were similar between the full-length and C-terminally truncated ASR, implying similar protein structural changes for the L state. The ASR_L_ minus ASR spectra were essentially similar to those of BR, but a unique spectral feature was observed in the carboxylic C=O stretching region. The bands at 1,722(+) and 1,703(−) cm^-1^ were observed at pH 5, which was reduced at pH 7 and disappeared at pH 9. The mutation study successfully assigned the bands to the C=O stretch of Glu36. Interestingly, Glu36 is located at the cytoplasmic side, and the distance from the retinal Schiff base is about 20 Å (Figure 31). We also observed pH-dependent frequency change of a water stretching vibration, which is located near Glu36.

As shown in this review article, ASR exhibits unique photoreaction properties. They are very different from those of other archaeal-type rhodopsins, and optimized for the photochromism sensor. The X-ray crystal structure of ASR reported the similar protein architecture characteristic of archaeal-type rhodopsins (Figure 4). Why is such functional optimization achieved in ASR? This is still a question that should be answered in future. Nevertheless, we suggest an important role of internal water molecules, particularly (i) at the Schiff base, and (ii) in the cytoplasmic side.

**Figure 40.**
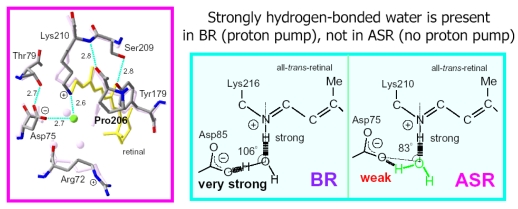
X-ray crystallographic structures of the Schiff base region of ASR and BR (Left). Schematic drawing of hydrogen bonds of the water molecule locating between the protonated Schiff base and its counterion (Right). This figure is reprinted with permission from TOC of Furutani *et al* [20]. Copyright 2005 American Chemical Society.

Like BR, ASR has a bridged water molecule between the Schiff base and Asp75 (Figure 4). However, Section 2 clearly showed the absence of strongly hydrogen-bonded water molecules in ASR. The reason for the lack of strongly hydrogen bonded water molecules in ASR was explained by the difference in the geometry of the hydrogen bond. Figure 40 shows that the N-Owater-OAsp75 (the Schiff base nitrogen, the water oxygen, and the oxygen of Asp75, respectively) angle in ASR is 83°. The corresponding N-Owater-OAsp85 angle in BR is 106° (Figure 40). As the consequence, if the water oxygen fully accepts the hydrogen bond of the Schiff base, the O-H group of water points toward the oxygen of Asp85 in BR, but not toward that of Asp75 in ASR (Figure 40). Such a small difference in angle possibly determines the hydrogen bonding strength of water molecules. On the basis of our FTIR studies of BR mutants and other rhodopsins, we have found the strong correlation between strongly hydrogen bonded water molecules and proton pump activity. It is likely that the strong hydrogen bond of the bridged water molecule is essential for the proton-pumping function of rhodopsins, presumably because of light-energy storage through transient weakening by retinal photoisomerization. Photo-cyclic reaction is important for the efficient proton pumping in rhodopsins. Thus, weak hydrogen bond of the Schiff base water is one of the key characters in ASR.

**Figure 41.**
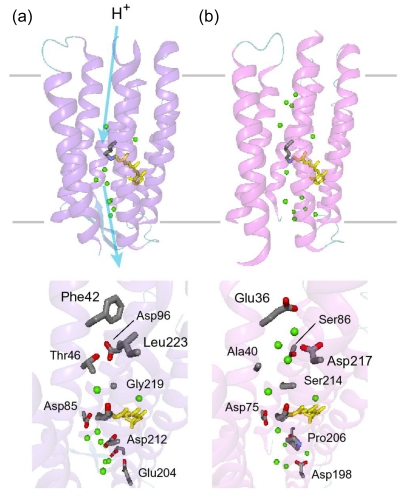
X-ray crystallographic structures of BR [7] (a) and ASR [16] (b). Top and bottom panels represent views from the membrane plane and the cytoplasmic side, respectively. In the top panel, top and bottom regions correspond to the cytoplasmic and extracellular sides, respectively. The retinal chromophore is colored yellow, and green spheres represent internal water molecules.

It should be however noted that many other rhodopsins do not possess strongly hydrogen-bonded water molecules, but photochromic reaction can be only seen for ASR. In this sense, polar cytoplasmic domain of ASR may play important role. Figure 41 shows that the hydrophobicity is different between the cytoplasmic and extracellular domains of BR. The cytoplasmic domain is highly hydrophobic, whereas the extracellular domain is composed of charged and polar amino acids that form a hydrogen-bonding network. Figure 41a shows the presence of 7-8 water molecules in the extracellular domain, but only 2 water molecules in the cytoplasmic domain. Such an asymmetric hydrogen-bonded network could be the reason of unidirectional proton transport in BR, where the proton transfer to the extracellular side occurs in 10^-5^ seconds, followed by reprotonation through a transiently formed proton pathway in the cytoplasmic domain on a slower timescale (10^-4^–10^-3^ seconds). The X-ray crystallographic structure of ASR has a similar α-helical arrangement to that of BR, but a very different hydrogen-bonded network. Figure 41b shows that in ASR both extracellular and cytoplasmic domains contain 5 water molecules, and form hydrogen-bonded networks. Such water-containing hydrogen-bonding network in the cytoplasmic region may be important in the photochromic reaction of ASR. Further study will reveal the unique reaction mechanism of the novel and interesting rhodopsin.

## Figures and Tables

**Figure 39.**
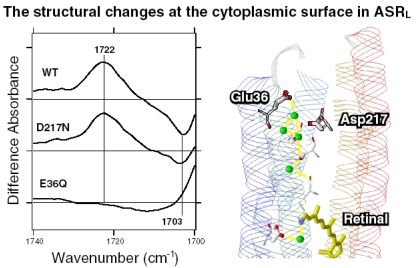
The structural changes of ASR in L-intermediate. (Left) The ASR_L_ minus ASR infrared spectra in protonated carboxylic acid region. (Right) The structure of ASR in cytoplasmic surface. Yellow broken lines indicates hydrogen bond, green spheres are water molecules. This figure is reprinted with permission from TOC of Kawanabe *et al* [77]. Copyright 2008 American Chemical Society.
